# Human caspase-4 and caspase-5 regulate the one-step non-canonical inflammasome activation in monocytes

**DOI:** 10.1038/ncomms9761

**Published:** 2015-10-28

**Authors:** Elena Viganò, Catherine Emma Diamond, Roberto Spreafico, Akhila Balachander, Radoslaw M. Sobota, Alessandra Mortellaro

**Affiliations:** 1 Singapore Immunology Network (SIgN), Agency for Science, Technology and Research (A*STAR), Singapore. 8A Biomedical Grove, #04-06 Immunos, Singapore 138648, Singapore; 2 University of Milano-Bicocca, PhD program in Translational and Molecular Medicine (DIMET), Ospedale San Gerardo, Via Pergolesi 33, Monza (MB) 20900, Italy; 3 Faculty of Life Sciences, The University of Manchester, Carys Bannister Building, Dover Street, Manchester M13 9PT, UK

## Abstract

Monocytes promote the early host response to infection releasing key pro-inflammatory cytokines, such as IL-1β. The biologically inactive IL-1β precursor is processed to active form by inflammasomes, multi-protein complexes activating caspase-1. Human monocytes exhibit an unconventional one-step pathway of inflammasome activation in response to lipopolysaccharide (LPS) alone. Although this lineage-restricted mechanism is likely to contribute to the pathology of endotoxin shock, signalling pathways regulating this mechanism are currently unknown. Here we report that caspase-4 and caspase-5 mediate IL-1α and IL-1β release from human monocytes after LPS stimulation. Although caspase-4 remains uncleaved, caspase-5 undergoes rapid processing upon LPS treatment. We also identify an additional caspase-5 cleavage product in LPS-stimulated monocytes, which correlates with IL-1 secretion. This one-step pathway requires Syk activity and Ca^2+^ flux instigated by CD14/TLR4-mediated LPS internalization. Identification of caspase-4/5 as the key determinants of one-step inflammasome activation in human monocytes provides potential targets for therapeutic intervention in endotoxin shock.

## Abstract (web summary)

Human monocytes exhibit an unconventional one-step pathway of inflammasome activation and IL-1 release in response to LPS. Here the authors show that it is mediated by caspases 4 and 5, and characterize caspase 5 cleavage, Syk and calcium signalling as key mediators of this pathway.

## Graphical abstract

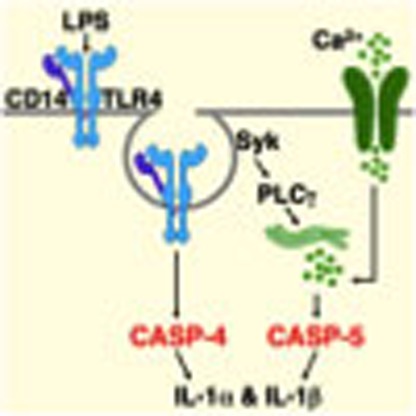

Monocytes are key mediators of early host responses to microbial infections^1^. The steady state recruitment of blood monocytes into peripheral tissues and local differentiation of these cells *in situ* is accelerated during microbial infection, allowing the rapid replenishment of macrophage and dendritic cell (DC) populations at the site of bacterial invasion. In infected tissues, monocytes, macrophages and DCs act in concert to eradicate pathogens by internalizing bacteria, producing anti-microbial factors, releasing pro-inflammatory cytokines, and promoting adaptive immune responses^2^. Early monocyte recruitment is therefore essential for effective host protection against diverse microbial infections^3^,^4^,^5^. If not rapidly contained, severe infections may involve translocation of viable bacteria into the blood, where they can elicit an acute severe inflammatory response that leads to sepsis (also known as systemic inflammatory response syndrome, or SIRS)^6^. Although human sepsis confers a high burden of morbidity and mortality around the world and causes millions of deaths each year, the mechanisms by which human monocytes are activated by microbial antigens and promote SIRS during acute infections are still not fully understood.

Monocytes expand rapidly in the circulation of sepsis patients and produce large quantities of pro-inflammatory cytokines including IL-1α/β, TNFα and IL-6 in response to bacterial lipopolysaccharide (LPS)^7^,^8^,^9^. Unlike tissue-resident cells such as macrophages, circulating monocytes exhibit extremely rapid and robust responses to bacterial infections^1^,^10^,^11^. Accordingly, monocyte differentiation into macrophages is typically accompanied by a decrease in capacity to secrete pro-inflammatory cytokines upon LPS exposure^12^,^13^,^14^. Both monocytes and macrophages can produce the potent ‘acute phase' cytokine IL-1β, which is a key regulator of the immune response to tissue injury and microbial invasion^15^. Although IL-1β exerts potent host protective effects during bacterial infection, excess production of this cytokine is also associated with septic shock, deregulated inflammation, and autoimmune pathologies^15^.

IL-1β cytokine is synthesized as an inactive precursor molecule (pro-IL-1β), which must be converted into a mature form to become functional. Processing of pro-IL-1β is mediated by the enzyme caspase-1, which is activated by multi-protein ‘inflammasome' complexes. Inflammasomes include distinct NOD-like receptors, such as NLRP1, NLRP3, NLRP6 and NLRC4 (ref. 16, 17, 18, 19). In macrophages and DCs, activation of the NLRP3 inflammasome requires two separate signals; (i) a priming stimulus, such as LPS-induced activation of the NF-κB pathway that increases transcription of NLRP3 and pro-IL-1β (ref. 20), and (ii) a second activation signal, that induces assembly of the inflammasome complex via the recruitment of pro-caspase-1 and adaptor molecule apoptosis-associated speck-like protein containing a caspase-recruitment domain (ASC). Only when both of these events occur in parallel, macrophages and DCs can initiate proteolytic cleavage of caspase-1 and mediate maturation of pro-IL-1β into active cytokine^21^. In contrast, monocytes exhibit a distinctive one-step pathway of inflammasome activation that can induce robust IL-1β release in response to TLR4 stimulation alone without the need for a secondary signal^14^. Despite a likely central role for this monocyte-specific mechanism of inflammasome activation in the pathogenesis of human sepsis, the key mediators of this pathway in LPS-stimulated human monocytes are currently unknown.

Despite reports that LPS sensitivity is up to three orders of magnitude lower in mice than in humans^22^, the majority of previous data on mechanisms of inflammasome activation come from studies in murine macrophages and DCs, which cannot be easily extrapolated for therapeutic applications in human patients. Indeed, human monocytes are thought to be key mediators of the pathogenesis of sepsis, but the molecular mechanisms by which LPS activates the inflammasome in these TLR4-expressing cells remain unknown. In the current report, we have identified a novel caspase-4/5-dependent pathway that enables human monocytes to rapidly release IL-1α/β following treatment with LPS. Therapeutic targeting of this novel pathway may prove beneficial in acute and chronic human inflammatory disorders.

## Results

### Human monocytes release IL-1β in response to LPS alone

**Figure 1:**
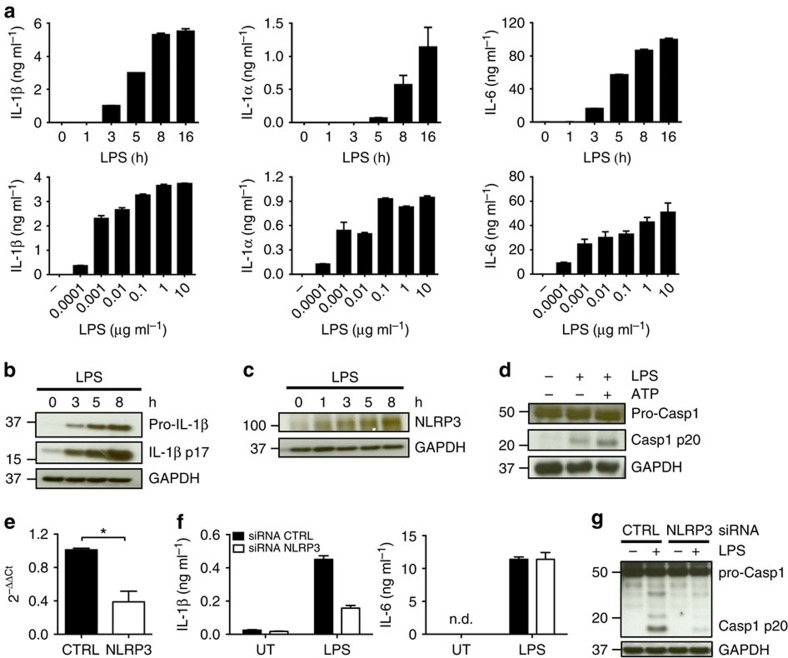
LPS acts as both a priming signal and NLRP3 activator in human monocytes. (**a**) Human monocytes were stimulated or not with 10 ng ml^−1^ LPS for 1–16 h (upper panels) or were activated for 5 h duration with 0.0001–10 μg ml^−1^ LPS (lower panels). Responses were analysed by ELISA measurement of IL-1α, IL-1β and IL-6 release into the supernatants. (**b**,**c**) Western blot analysis of pro-IL-1β, active IL-1β p17 (**b**) and NLRP3 (**c**) in monocytes stimulated with LPS for the indicated times. Pro-IL-1β, IL-1β p17 and NLRP3 expression were assessed in total cell lysates. (**d**) Western blot analysis of pro-caspase-1, caspase-1 p20 and GAPDH in monocytes cultured in medium, LPS alone, or LPS in combination with ATP. (**e**) Quantitative real-time PCR was used to validate the siRNA-mediated decrease in the level of NLRP3 transcripts in monocytes. (**f**) IL-1β and IL-6 release from monocytes in which NLRP3 expression was reduced using siRNA. n.d., not detected. (**g**) Caspase-1 processing in monocytes treated with control siRNA (CTRL) or NLRP3 siRNA and stimulated with LPS was evaluated by western blot. Blots are representative of three independent experiments. Graphs in a and f show the mean±s.d. of triplicate wells and are representative of three or more independent experiments. Graph e shows the mean±s.d. of three independent experiments. A one sample *t*-test has been performed to evaluate the significance. *P value<0.05.

LPS stimulation alone was sufficient to induce secretion of pro-inflammatory cytokines IL-1α, IL-1β and IL-6 (Fig. 1a), rapid synthesis of the pro-IL-1β precursor (Fig. 1b), upregulation of NLRP3 expression (Fig. 1c), and activation of caspase-1 (Fig. 1d) in human monocytes. Addition of the conventional NLRP3-activating signal ATP potentiated further caspase-1 cleavage in LPS-stimulated human monocytes (Fig. 1d), but this additional stimulus was not required to elicit substantial IL-1β release (Fig. 1a). To assess the role of NLRP3 in the release of IL-1β and the processing of caspase-1 in response to LPS stimulation, NLRP3 expression was knocked down in monocytes using specific siRNA duplexes (Fig. 1e). NLRP3 downregulation caused a significant attenuation of IL-1β secretion (Fig. 1f) and caspase-1 processing (Fig. 1g) compared with the control siRNA. However, the release of IL-6, an inflammasome-independent cytokine, remained unchanged (Fig. 1f). These data indicate that LPS exposure alone is capable of activating the NLRP3 inflammasome in human monocytes, leading to increased processing of pro-IL-1β precursor and subsequent release of active IL-1β p17.

The mechanism of NLRP3 inflammasome activation in monocytes differs significantly from that observed in fully differentiated macrophages and DCs. Accordingly, while LPS exposure alone was able to induce marked IL-6 production by human macrophages and DCs (Supplementary Fig. 1a,c), this stimulus was unable to promote caspase-1 processing (Supplementary Fig. 1b,d), and IL-1β secretion (Supplementary Fig. 1a,c) except in the presence of ATP. Human monocytes therefore exhibit an alternative pathway of NLRP3 inflammasome activation, which is mechanistically distinct from the currently established model described primarily in macrophages and DCs.

### LPS induces processing of caspase-5 in human monocytes

The murine inflammatory caspase-1 and -11 are essential mediators of septic shock *in vivo*^23^,^24^. Recent studies have also identified caspase-11 as a key regulator of the non-canonical inflammasome activation responsible for IL-1α release in response to intracellular LPS and Gram-negative bacteria, as well as a crucial inducer of inflammasome-associated cell death^25^,^26^,^27^.

The putative human orthologues of murine caspase-11 are caspase-4 and -5, based on amino acid sequence. Current understanding of the expression and activation of caspase-4/5, as well as of the mechanisms regulating caspase-4/5-mediated activation of inflammasome activation, is still limited. Therefore, we decided to investigate the role of these caspases in the IL-1 pathway in LPS-stimulated monocytes.

**Figure 2:**
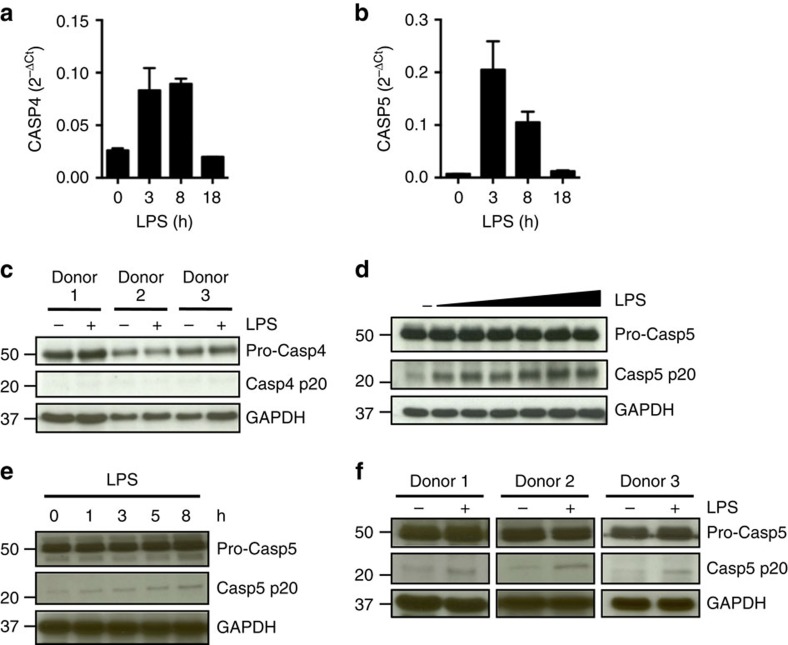
LPS triggers the processing of caspase-5, but not caspase-4, in human monocytes. (**a**,**b**) Caspase-4 and -5 mRNA expression in monocytes treated with LPS (10 ng ml^−1^) for the indicated times was measured by quantitative real-time PCR. (**c**) Western blot analysis of caspase-4 (pro-form and cleaved p20) in cell lysates of monocytes stimulated for 5 h with 10 ng ml^−1^ LPS. (**d**–**f**) Western blot analyses of pro-caspase-5 and caspase-5 p20 in monocytes stimulated with increasing LPS concentrations (0.0001–10 μg ml^−1^) (**d**) or a fixed concentration of LPS (10 ng ml^−1^) for variable duration (**e**). (**f**) Caspase-5 processing was assessed by western blot in lysates of monocytes from three representative donors following LPS (10 ng ml^−1^) for 5 h. GAPDH was used as the loading control. Blots are representative of three independent experiments.

We first evaluated the expression of caspase-4 and -5 in human monocytes at steady-state and after LPS treatment. Caspase-4 and -5 mRNA expression was increased after 3 h post stimulation with LPS diminishing to basal levels after 18 h (Fig. 2a,b), while protein levels remained unchanged (Fig. 2c–f).

In contrast to caspase-4 regulation, LPS stimulation of monocytes induced processing of pro-caspase-5 as reflected by the appearance of its p20 subunit in a dose- and time-dependent manner (Fig. 2d,e). Caspase-5 processing was consistent across different donors (Fig. 2f). Since caspase-5, but not caspase-4, underwent processing, LPS seems to be an inducer of unconventional caspase-5 processing in human monocytes.

### LPS-mediated IL-1 release depends on caspase-4/5 in monocytes

**Figure 3:**
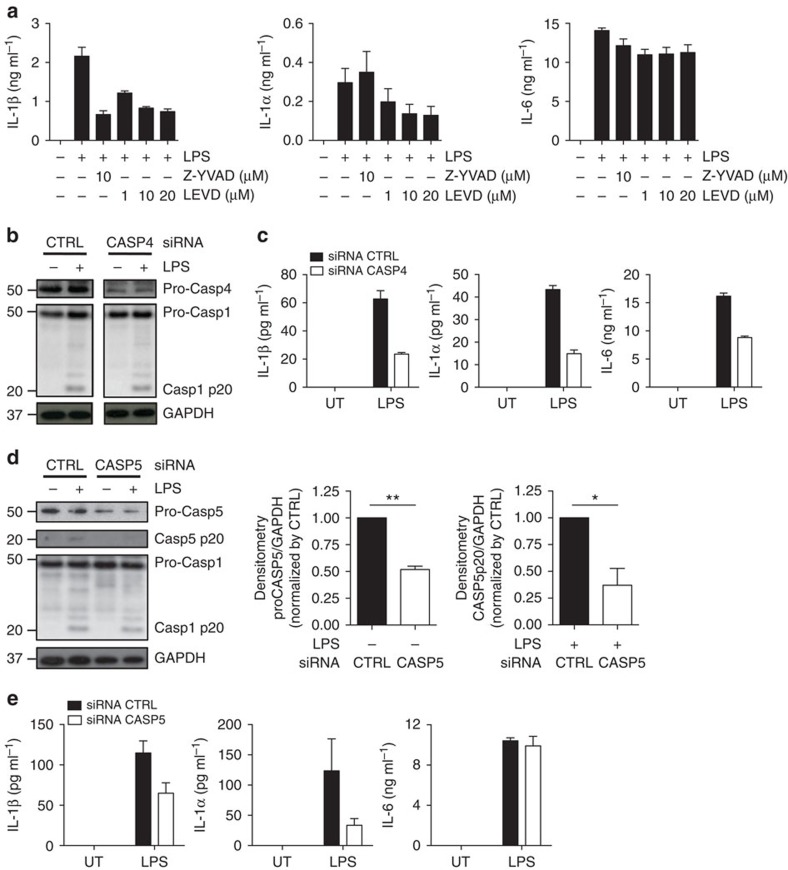
Caspase-4 and -5 are required for IL-1α and IL-1β release. (**a**) Monocytes were treated with either Z-YVAD-fmk (caspase-1 inhibitor) or LEVD-fmk (caspase-4/5 inhibitor) for 1 h before LPS stimulation. Culture supernatants were collected after 5 h and secretion of IL-1α, IL-1β and IL-6 was determined by ELISA. (**b**,**e**) Silencing of caspase-4 (**b**) or caspase-5 (**d**) was assessed by western blot in siRNA-treated monocytes stimulated with LPS. GAPDH was used as loading control. Densitometry analysis of pro-caspase-5 and caspase-5 p20 was also shown. (**c**,**e**) Cytokine release from siRNA-treated monocytes following LPS stimulation was evaluated by ELISA. Blots are representative of three independent experiments. Graphs show the mean±s.d. of triplicate wells, and are representative of three or more independent experiments. *P value<0.05, **P value<0.01.

We next sought to determine whether caspase-4 and/or -5 regulate the secretion of inflammasome-activated IL-1α and IL-1β in human monocytes following exposure to LPS. We first determined whether caspase-4/5 activity was required for cytokine release. Monocytes were pre-treated with the LEVD-fmk inhibitor, which blocks the activity of both caspase-4 and -5 before LPS stimulation. The release of IL-1α and IL-1β was significantly impaired when LEVD-fmk inhibitor was added to the culture, whereas the caspase-1 inhibitor Z-YVAD-fmk suppressed the release of IL-1β only (Fig. 3a). LPS-induced IL-6 release from monocytes remained stable in the presence of the LEVD-fmk inhibitor (Fig. 3a), indicating that the inhibitor was not toxic.

To further ascertain the individual role for caspase-4 and -5 in IL-1α and IL-1β release we used siRNAs to knockdown the expression of these caspases in monocytes. The caspase-4 siRNA duplexes effectively suppressed caspase-4 mRNA and protein (Supplementary Fig. 2a and Fig. 3b), but not caspase-1 or -5 mRNA (Supplementary Fig. 2a). Silencing of caspase-4 significantly suppressed the release of IL-1α and IL-1β, as well as IL-6, from LPS-treated monocytes (Fig. 3c). These results, obtained from four independent donors (Supplementary Fig. 2b), confirm the role of caspase-4 in LPS-mediated release of cytokines, including those that do not require inflammasome activation, such as IL-6. The fact that IL-6 secretion was also impaired in caspase-4-defective monocytes is indicative of a possible involvement of caspase-4 in NF-κB-mediated gene transcription, as reported by Lakshmanan *et al*.^28^ in THP1 cells. However, our data obtained by using the pharmacological inhibitor indicate that IL-6 release does not depend on caspase-4 activity.

Upon siRNA-mediated silencing, caspase-5 expression was substantially reduced in human monocytes at both protein (Fig. 3d) and mRNA levels (Supplementary Fig. 2c), while expression of caspase-1 and caspase-4 remained unaltered. Pro-caspase-5 silencing coincided also with casp-5 p20 reduction, as assessed by western blot (Fig. 3d), and considerable suppression of both IL-1α and IL-1β from monocytes after LPS stimulation (Fig. 3e). In contrast to caspase-4 silencing, there was no significant effect of caspase-5 siRNA duplexes on IL-6 production (Fig. 3e). These results have been confirmed in monocytes from three independent donors (Supplementary Fig. 2d) using either single or pooled siRNA duplexes (Supplementary Fig. 2e,f).

NLRP3 is an essential effector of caspase-1 activation instigated by canonical stimuli^17^. Since, we have previously shown that LPS induced caspase-1 processing in a NLRP3-dependent fashion (Fig. 1g), we then sought to determine whether non-canonical caspase-4 and -5 contribute to caspase-1 activation following LPS stimulation. We found that neither caspase-4 nor caspase-5 significantly altered caspase-1 processing in LPS-stimulated monocytes (Fig. 3b,d). This result indicates that the NLRP3-dependent pathway is the major contributor to caspase-1 activation.

Caspase-4 has recently been found to be involved in the induction of cell death^29^,^30^. To determine whether caspase-5 is similarly involved in cell death induced by LPS, we stimulated monocytes with LPS for 5 and 24 h and measured the release of lactate dehydrogenase (LDH) in cell-free supernatants. Untreated and LPS-stimulated monocytes released comparable levels of LDH (Supplementary Fig. 3a). These results, confirmed by annexin V, 7AAD staining and flow cytometry (Supplementary Fig. 3b), indicate that monocytes are refractory to LPS-induced cell death, as also reported by others^31^,^32^.

### IFNAR pathway is dispensable for caspase-5 processing

**Figure 4:**
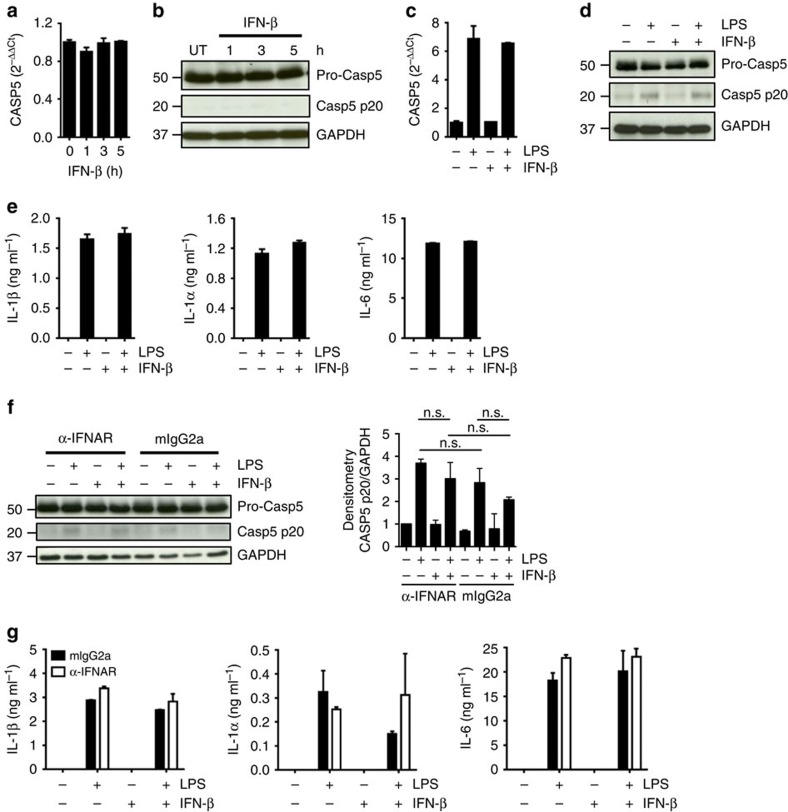
Type-I IFNs are dispensable for caspase-5 activation in human monocytes. (**a**–**d**) Quantitative real-time PCR (**a**,**c**) and western blot analysis (**b**,**d**) of caspase-5 in monocytes treated for different times with IFN-β alone (**a**,**b**) or in combination with LPS (**c**,**d**). (**e**) IL-1α, IL-1β and IL-6 release by monocytes treated with LPS alone or in combination with IFN-β for 5 h. (**f**,**g**) Monocytes were pre-treated with a neutralizing anti-IFNAR (α-IFNAR) antibody or isotype-matched control antibody (mIgG2a) and then stimulated with IFN-β alone, LPS alone, or IFN-β and LPS together for 5 h. (**f**) Caspase-5 processing was assessed by western blot (left). Densitometry analysis of caspase-5 p20 was also shown (right). n.s., non significant. (**g**) Cytokine release was assessed by ELISA. Blots are representative of two or more independent experiments. Graphs show the mean±s.d. of triplicate wells and are representative of three independent experiments.

Because LPS elicited caspase-5, but not caspase-4 processing we examined which are the upstream mechanisms regulating caspase-5 activation. Previous studies have reported that caspase-11 expression in mouse macrophages requires type-I interferons (IFNs)^33^,^34^,^35^. We first assessed whether autocrine IFN-β signalling could contribute to the activation of caspase-5, as human monocytes secrete high levels of IFN-β upon LPS stimulation. High doses of recombinant IFN-β, either alone or in combination with LPS, induced marked expression of interferon-stimulated genes (ISGs) (Supplementary Fig. 4a), but failed to increase either the expression or processing of caspase-5 (Fig. 4a–d). Accordingly, LPS-induced monocyte release of IL-1α, IL-1β and IL-6 was unaffected by addition of exogenous IFN-β cytokine (Fig. 4e). Consistent with these data, antibody neutralization of the IFN-α/β receptor (IFNAR) potently reduced ISG expression (Supplementary Fig. 4b), but did not reduce caspase-5 processing induced by LPS (Fig. 4f, lanes 2 versus 6), or cytokine release (Fig. 4g). These data indicate that the IFNAR pathway does not trigger caspase-5 activation in monocytes exposed to LPS, highlighting significant divergence in the regulation of the human and murine orthologues.

### LPS internalization is required for caspase-5 activation

Our data indicated that caspase-5 activation in LPS-stimulated human monocytes employs distinct mechanisms from those that activate murine caspase-11. We therefore sought to identify the signalling events that mediate caspase-5 processing and IL-1α/β release from human cells. LPS is sensed by immune cells through Toll-like receptor 4 (TLR4). To test whether TLR4 is required for caspase-5 processing, monocytes were pre-incubated with LPS from *Rhodobacter sphaeroides* (LPS-RS) before LPS stimulation. LPS-RS is a potent antagonist of LPS from pathogenic bacteria, and suppresses TLR4 signalling in human cells^36^. We found that blocking of TLR4 using LPS-RS effectively suppressed LPS-induced processing of caspase-5 (Supplementary Fig. 5a).

LPS comprises the lipid A moiety, the core oligosaccharide and the O-antigen, a polysaccharide of variable length^37^. Although LPS mutants activate TLR4, they elicit different innate immune responses. In particular, the LPS-Re mutant, which lacks the saccharidic O-chain, and the lipid A moiety, activate the MyD88 pathway independent of the TLR4 co-receptor CD14 (ref. 38). We therefore tested the ability of LPS-Re mutant or lipid A to induce caspase-5 processing. We found that processing of caspase-5 p20 was significantly reduced in monocytes stimulated with lipid A, but not LPS-Re, which can act through CD14, compared with intact LPS (Supplementary Fig. 5b). These results indicate that TLR4 signalling is required for caspase-5 processing and suggest that CD14 may be partly involved.

One of the first events following LPS-TLR4 engagement is the internalization of the receptor-ligand complex into the endosomal compartments^39^. LPS-TLR4 delivery to endosomes requires the TLR4 co-receptor CD14 and the tyrosine kinase Syk^40^,^41^. We therefore hypothesized that caspase-5 activation may occur by a physiological process of LPS-induced CD14/TLR4 internalization mediated by Syk. We found that upon LPS-stimulation of monocytes the CD14/TLR4 complexes localized to Rab5^+^ early endosomes through a process that requires dynamin. Indeed, pre-treatment of monocytes with the specific dynamin inhibitor dynasore prevented CD14/TLR4 internalization (Supplementary Fig. 6a,b). Moreover, analysis of endotoxin distribution revealed that LPS was localized to the plasma membrane at a very early time point (1 min) and was then rapidly internalized (Supplementary Fig. 6c). Surprisingly, LPS internalization at later time points (>1 h) did not result in co-localization with either early or late endocytic markers, and instead exhibited a diffuse distribution suggestive of cytosolic localization (Supplementary Fig. 6d).

**Figure 5:**
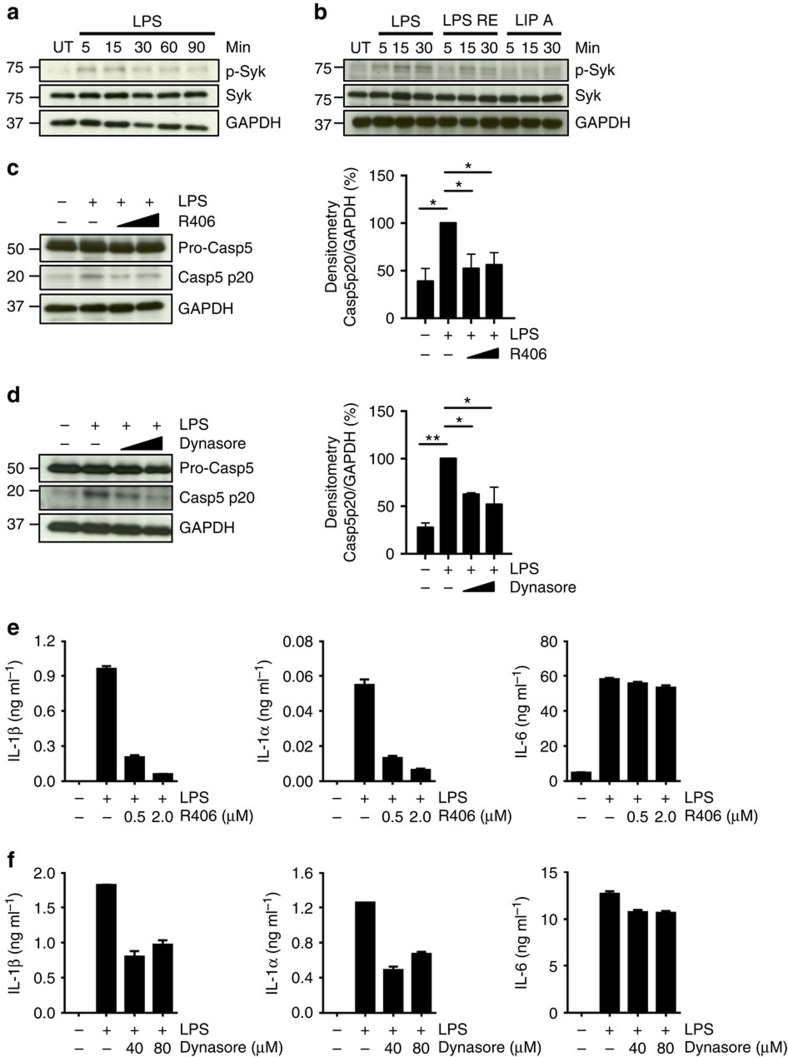
Syk kinase and Syk-mediated endocytosis of LPS trigger caspase-5 processing and cytokine release. (**a**,**b**) Western blot analysis of p-Syk (Tyr 525–526) or total Syk in monocytes that were either left untreated (UT) or stimulated with LPS, LPS-RE or lipid A for the indicated time-points. (**c**,**d**) Western blot analysis of caspase-5 and caspase-5 p20 in monocytes pre-treated for 1 h with the Syk inhibitor R406 (0.5–2 μM) (**c**) or the dynamin inhibitor dynasore (40–80 μM) (**d**) prior stimulation with LPS for 5 h. Blots are representative of three independent experiments. Densitometry analyses of caspase5 p20 are also shown (right). (**e**,**f**) Release of IL-1α, IL-1β and IL-6 from monocytes pre-treated with R406 (**e**) or dynasore (**f**) before LPS stimulation. Graphs (**e**,**f**) show the mean±s.d. of triplicate wells and are representative of three independent experiments. Graphs (**c**,**d**) (right) show the mean±s.d. of three independent experiments and significance evaluated using a one sample *t*-test. *P value<0.05, **P value<0.01.

We then tested the requirement for Syk in mediating this process. Monocyte stimulation with LPS induced rapid phosphorylation of Syk in human monocytes, which was significantly diminished in LPS-Re- and lipid A-treated monocytes (Fig. 5a,b). Pharmacological inhibition of Syk activity using R406 disrupted CD14/TLR4 internalization (Supplementary Fig. 7a,b). Consistent with our hypothesis, disruption of LPS endocytosis by interfering with Syk or dynamin activity significantly reduced caspase-5 cleavage (Fig. 5c,d), and consequently impaired the release of IL-1β and IL-1α (Fig. 5e,f). Release of IL-6, which requires TLR4 signalling from the plasma membrane, proceeded normally in dynasore- and R406-treated monocytes (Fig. 5e,f). These data demonstrate that LPS endocytosis is both necessary and sufficient to induce caspase-5 activation and release of IL-1β and IL-1α from human monocytes.

### Caspase-5 activation depends on Ca^2+^ mobilization

**Figure 6:**
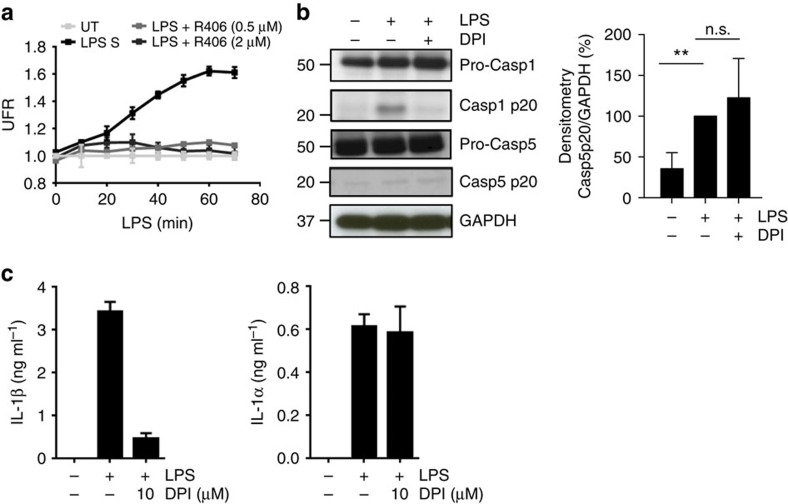
Caspase-5 activation is independent of ROS production. (**a**) ROS production was measured in monocytes pre-treated with the Syk inhibitor R406 before LPS stimulation. (**b**,**c**) Monocytes were pretreated with DPI inhibitor before LPS stimulation. Caspase-1 and caspase-5 activation (**b**, left) were assessed by western blot. Blot is representative of four independent experiments. Densitometry analysis of caspase-5 p20 is also shown as mean±s.d. of four independent experiments and significance evaluated using a one sample *t*-test (**b**, right). (**c**) IL-1α and IL-1β release were assessed by ELISA. Graphs (**a**,**c**) show the mean±s.d. of triplicate wells and are representative of three independent experiments. **P value<0.01, n.s. not significant.

In addition to its role in CD14/TLR4 endocytosis, Syk operates downstream of lectin receptors to promote synthesis of pro-IL-1β and generation of reactive oxygen species (ROS)^42^. ROS production in turn leads to NLRP3 inflammasome activation and release of mature IL-1β cytokine^42^. We therefore examined whether ROS contribute to LPS-induced activation of caspase-5 in human monocytes. LPS exposure robustly increased monocyte production of ROS, which was abrogated by inhibition of Syk activity (Fig. 6a). Intriguingly, the ROS inhibitor DPI efficiently suppressed caspase-1 activity, but did not impair processing of caspase-5 (Fig. 6b). Pharmacological inhibition of ROS synthesis also reduced monocyte secretion of IL-1β, but not IL-1α (Fig. 6c). These data indicate that ROS are dispensable for caspase-5 activation and IL-1α release, suggesting that additional mediators of this pathway may also be required.

**Figure 7:**
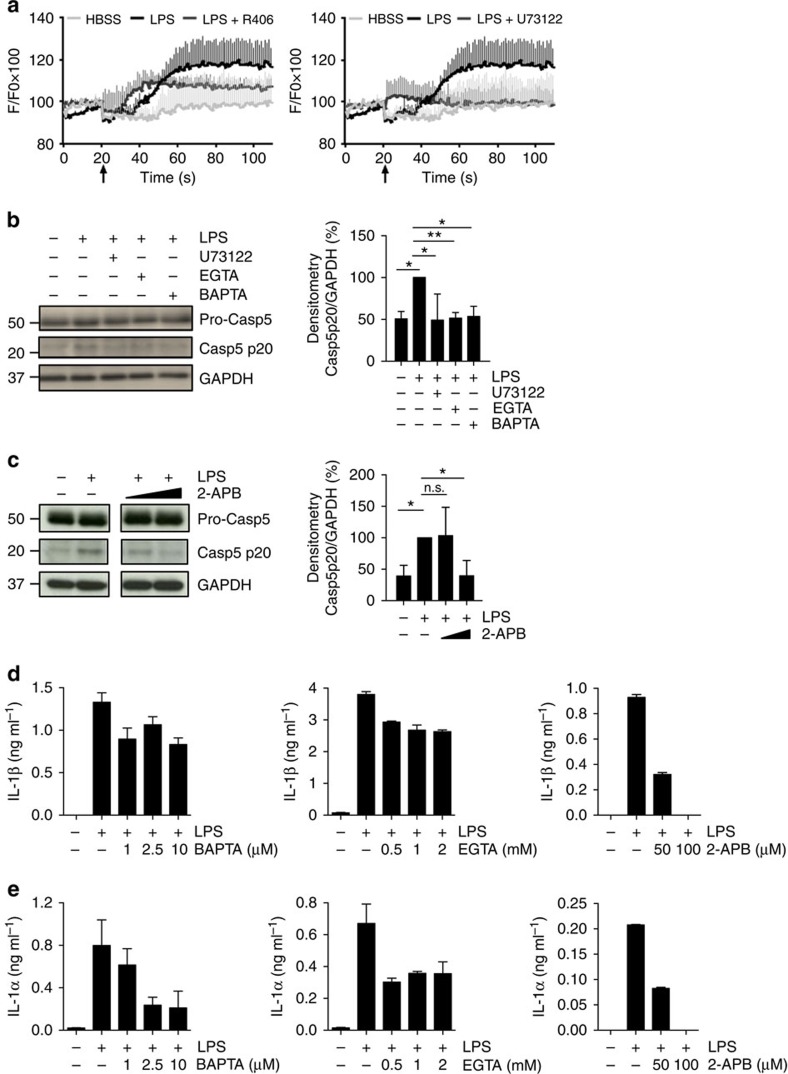
Caspase-5 activation depends on Ca^2+^ mobilization. Monocytes were pre-treated with the Syk inhibitor R406 (**a**), the PLCγ inhibitor U73122 (**a**,**b**) the Ca^2+^ chelators EGTA (0.5 and 2 mM, membrane non-permeant) or BAPTA (2.5 and 10 μM, membrane permeant) (**b**,**d**,**e**) and the IP3 receptor antagonist 2-APB (50 and 100 μM) (**c**–**e**) before LPS stimulation. Responses were analysed by measuring Ca^2+^ mobilization (**a**), activation of caspase-5 (**b**,**c**) and release of IL-1α and IL-1β (**d**,**e**). Blots are representative of three independent experiments. Densitometry analysis of caspase-5 p20 was also shown in (**b**,**c**) as mean±s.d. of three independent experiments. Significance is evaluated using the one sample *t*-test. Arrows in panel (**a**) indicate the time of HBSS or LPS addition. Graphs (**a**,**d**,**e**) show the mean±s.d. of triplicate wells and are representative of three independent experiments. *P value<0.05,**P value<0.01, n.s. not significant.

Syk also plays an essential role in the regulation of PLCγ-mediated Ca^2+^ mobilization. PLCγ activation mediated by Syk generates the formation of inositol 1,4,5-trisphosphate (IP3), which upon binding to its receptor in the endoplasmic reticulum provokes Ca^2+^ release into the cytosol^43^,^44^. Therefore, we assessed the role of Ca^2+^ during the one-step inflammasome activation triggered by LPS. We first confirmed that inhibition of Syk and PLCγ activity abrogated Ca^2+^ flux in LPS-stimulated monocytes (Fig. 7a). To assess the role of Ca^2+^ as a possible second messenger for caspase-5 activation, monocytes were pre-treated with a cell-permeable intracellular Ca^2+^ chelator (BAPTA/AM), an extracellular Ca^2+^ chelator (EGTA), or a permeable IP3 receptor antagonist (2-APB), before stimulation with LPS. We observed that caspase-5 processing (Fig. 7b,c), as well as IL-1β and IL-1α release (Fig. 7d,e) was dependent on Ca^2+^ mobilization.

Finally, we investigated whether the inability of macrophages and DCs to engage the unconventional one-step inflammasome pathway in response to endotoxin exposure, could be because of these cells exhibiting different responses to LPS. Monocytes express high levels of CD14 but when differentiated *in vitro* to macrophages and DCs they become CD14 low/negative (Supplementary Fig. 8a). Although macrophages and DCs retain the ability to respond to LPS, releasing several inflammatory factors in response to stimulation, the lack of CD14 expression resulted in an inability to activate the Syk pathway (Supplementary Fig. 8b). Indeed, while LPS induced Syk phosphorylation in monocytes, it was unable to do so in macrophages and DCs (Supplementary Fig. 8b). Moreover, LPS failed to induce Ca^2+^ mobilization in macrophages and DCs differentiated *in vitro*, although these cells responded normally to ATP exposure (Supplementary Fig. 8c), demonstrating their capacity to mobilize Ca^2+^. Defective Ca^2+^ signalling in macrophages and DCs may underlie their inability to engage this pathway in response to LPS. Surprisingly, LPS induced caspase-5 cleavage generating the p20 subunit in macrophages and DCs (Supplementary Fig. 8d). Interestingly, in monocytes we consistently observed the presence of a processed form of caspase-5 of approximately 10–12 kDa, which was not detected in DCs and macrophages upon LPS and LPS/ATP stimulation (Supplementary Fig. 8d). This processed form of caspase-5 appeared over time and with increasing doses of LPS; its formation was inhibited by Syk, dynamin and PLCγ inhibitors, as well as by monocyte pre-treatment with intracellular (BAPTA/AM) or extracellular (EGTA) Ca^2+^ chelators, or IP3 receptor antagonist (Supplementary Fig. 9a–f). The presence of the 10–12 kDa processed form of caspase-5 showed the same pattern of caspase-5 p20 in all conditions (Supplementary Figs 9 and 11).

The 10–12 kDa processed form of caspase-5 does not represent the small p10 subunit of caspase-5 because the antibody used in our study only detects the large p20 subunit. We therefore undertook a proteomic approach to identify its origin. Caspase-5 protein was labelled for 1 h with tandem mass tags, which modify primary amines, including free N-terminus derived as a consequence of endopeptidase cleavage. This method enabled us to mark and recognize caspase-5-cleaved sites before trypsin digestion. Using mass spectrometry, we identified the DMESVLR peptide (corresponding to amino acids 240–246), which marks the central part of caspase-5 p20 (Supplementary Fig. 10). The DME motif retains the same physical and biochemical properties of the consensus cleavage sequence identified in caspase-1, which requires an aspartic acid (D) at position P1. This newly identified cleavage site is located at the center of the caspase-5 p20 subunit, generating two peptides with a molecular mass of approximately 10–12 kDa. One peptide contains the caspase-5 active site (cysteine 315 of isoform a), while the other contains the Leu146 that is recognized by the antibody (Supplementary Fig. 10a). These data suggest that the D137-R239 peptide could be derived from the processing of caspase-5 p20.

Taken together, these results suggest that Syk/PLCγ-induced Ca^2+^ flux in monocytes is required for the processing of caspase-5 p20 and its further cleavage into the D137-R239 peptide. This is consistent with activation of the unconventional inflammasome pathway in LPS-stimulated monocytes.

## Discussion

Monocytes are the most abundant CD14/TLR4-expressing cell type in the human circulation and play a crucial role in sepsis^1^,^7^. The identification of the molecular mechanism by which LPS induces monocyte activation therefore has important clinical implications. We report that caspase-5, as much as caspase-4, contributes to TLR4-mediated IL-1α/β release from human monocytes.

Based on protein sequence caspase-4 and -5 are classified as the inflammatory caspases most homologous to murine caspase-11. It is possible that distinct mechanisms of inflammasome activation may have evolved in different species and different cell types. At the moment there is no consensus on which of the two caspases represents the functional orthologue of murine caspase-11.

Caspase-4 has been associated with pyroptotic cell death in mono-myelocytic cells lines (THP1, U937) induced by intracellular LPS delivery^29^. In addition, transgenic expression of human caspase-4 in mice supported caspase-1 activation and enhanced IL-1β/IL-18 release in response to LPS^45^. A recent study showed that caspase-4 contributes to inflammasome activation and IL-1α, but not IL-1β release from human macrophages upon infection with Gram-negative bacteria^30^. We provided further evidence that although caspase-4 does not undergo processing, it mediates secretion of both IL-1α and IL-1β induced by LPS stimulation. These data, together with results published by Shi *et al*.^29^ indicate that caspase-4 precursor not only binds to LPS but is also functionally active. We found that LPS-induced release of IL-6 from caspase-4-deficient monocytes was also affected, suggesting that other cytokines and chemokines regulated by LPS-mediated NF-κB activation may also require caspase-4. Indeed, a previous study by Lakshmanan *et al*.^28^ reported that human caspase-4-deficient THP1 cells exhibited substantial reduction of LPS-induced secretion of CXCL8, CCL4, CCL20, and IL-1β. It was shown that LPS-induced NF-κB nuclear translocation and activation was dependent on caspase-4 through its interaction with TNFR-associated factor 6 (TRAF6).

Little is known about caspase-5, the mechanisms involved in its activation and physiological functions. Caspase-5 is primarily expressed in tissues that are in close contact with pathogens, such as in skin^46^,^47^. Caspase-5 has not previously been implicated in the host response to microbial products, since early studies suggested that this enzyme was in fact a component of the NLRP1 inflammasome^48^, and that it mainly contributed to the induction of apoptosis^49^,^50^. The specific activating stimuli and target substrates of caspase-5 had until now remained obscure. In our study, we have identified caspase-4 and -5 as being crucial downstream targets of LPS activation in human monocytes. We found that caspase-5 contributes to IL-1α and IL-1β, but not IL-6, release from LPS-stimulated monocytes.

Moreover, although both caspase-4 and -5 regulate IL-1β release from monocytes after LPS stimulation, our evidence indicates that NLRP3 remains the main player for caspase-1 activation. Further studies are needed to dissect the exclusive and overlapping roles of caspase-4 and -5, as well as their involvement in the canonical NLRP3 inflammasome activation.

Interestingly, in addition to the caspase-5 p20 subunit we identified an additional 10–12 kDa fragment using mass spectrometry analysis, which is derived from a cleavage site at the centre of the p20 subunit, containing the catalytic site. The presence of the 10–12 kDa processed form of caspase-5 strongly correlated with the ability of monocytes to activate the unconventional one-step inflammasome pathway and release IL-1α/β by LPS alone. Indeed, it is constantly present in LPS-stimulated monocytes (Supplementary Figs 9 and 11), but not in cells that are unable to activate the NLRP3 inflammasome by LPS alone [macrophages and DCs (Supplementary Fig. 8d)]. In consideration of the fact that the processed D137-R239 form of caspase-5 contains the catalytic site, it would be interesting in future studies to assess if its activity is conserved; although a lack of knowledge regarding the substrates of caspase-5 renders this task extremely difficult.

In mice, a non-canonical inflammasome pathway is regulated by caspase-11 activated by forced introduction of LPS into the cytoplasm via non-physiological means, such as cholera toxin B-mediated delivery, electroporation or lipofection^26^,^27^,^29^. This non-canonical inflammasome pathway is TLR4 independent and leads to the activation of the inflammatory caspase-11 which promotes pyroptosis and IL-1α release^26^,^27^,^29^. In our studies, exogenous LPS, via TLR4 signalling, triggers cytokine release but no cell death in monocytes. These results are consistent with previous reports showing that monocytes undergo apoptosis spontaneously upon culture, and that activating stimuli (that is, LPS, GM-CSF, M-CSF, TNFα and IL-1β) prolong monocyte survival *in vitro*^31^,^51^,^52^. Activation-mediated survival is physiologically essential for the functional integrity of monocytes enabling the cells to promptly respond to various insults (that is, microbes). A similar point of divergence between cytokine release and cell death induction by the inflammasomes has been recently described in murine neutrophils in response to Gram-negative bacteria^53^, and it would be interesting to investigate the possible contribution of caspase-11/4/5 in neutrophils. However, we cannot exclude that caspase-4 and/or caspase-5 might play a role in pyroptosis when a different cell system and/or trigger is used. Further studies will be necessary to clarify the role of caspase-4 and -5 in monocyte cell death.

It was shown that caspase-11, as well as human caspases-4 and -5, can directly sense intracellular LPS and trigger self-activation^29^. However, in these studies, the LPS stimulus was delivered via electroporation in macrophages pre-primed by poly (I:C) (TLR3 agonist) and did not occur via TLR4-mediated endocytic pathway. In our study, human monocytes were activated by extracellular addition of LPS, without the use of artificial methods to deliver LPS into the cytosol. Using this approach, we observed that LPS immediately localized to the plasma membrane and was rapidly internalized by monocytes via conventional endocytic pathways. LPS internalization was regulated by Syk and inhibition of this tyrosine kinase negatively impacted caspase-5 processing and IL-1α/β release by monocytes. The critical importance of physiological LPS uptake was also demonstrated by impaired monocyte activation after inhibition of endocytosis mediated by dynamin.

Before our study, Syk was already known to be involved in the activation of caspase-1 (ref. 42), but the novel role we identified for this enzyme in caspase-5-mediated activation has not previously been reported. Here, we showed that Syk-mediated Ca^2+^ flux via PLCγ/IP3 receptor was required for caspase-5 processing and cytokine release. Syk is activated by CD14 engagement by LPS^41^. Consistently, we found that only LPS moieties engaging CD14 activate Syk and caspase-5 processing in monocytes. It is tempting to speculate that only CD14-expressing cells can employ this one-step response to LPS. Human conventional monocytes express large amounts of CD14, but when these cells are cultured in the presence of GM-CSF/IL-4 or M-CSF, used to induce their differentiation into DCs and macrophages, respectively, they become CD14^low/neg^ (Supplementary Fig. 8a and refs 54, 55). This could be the reason why macrophages and DCs are unable to activate Syk and mobilize Ca^2+^ upon LPS exposure and hence, they could not release IL-1α/β unless a proper inflammasome activator, such as ATP, is provided.

In a previous study, it was concluded that human blood monocytes exhibit constitutive activation of caspase-1 and therefore do not require additional signals to respond to LPS^56^. On the contrary, here we report that caspase-1 remains uncleaved in resting monocytes and that genetic suppression of caspase-5 expression impaired the LPS-induced release of IL-1α and IL-1β by the NLRP3 inflammasome.

Not only are the cell types that employ this pathway distinct from one another, but also the mechanisms that generate the main products of this pathway, namely IL-1α and IL-1β. Previous data have already demonstrated that the mechanisms of IL-1α secretion and IL-1β release are distinct in murine cells^25^; here we report for the first time that this dichotomy also applies to human monocytes. We observed that IL-1α/β pathway divergence occurs downstream of Syk, likely at the level of ROS production and Ca^2+^ mobilization, since abrogation of the former impaired the secretion of IL-1β but not IL-1α. In contrast, inhibition of Ca^2+^ mobilization down-regulated the secretion of both cytokines.

A final consideration is that while the one-step inflammasome pathway, in both mice and human monocytes, needs caspase-1 activation, it also exhibits important species-specific differences in its regulation. Human caspase-4/5 are already expressed in resting monocytes, macrophages and DCs, whereas murine caspase-11 is expressed only upon induction by type-I IFN^34^,^35^. In contrast, human monocytes can activate caspase-5 in response to LPS and type-I IFN signalling was dispensable for enzyme activation.

In conclusion, our study describes a new molecular mechanism of IL-1α/β secretion that operates specifically in LPS-stimulated human monocytes with no requirement for additional external signals, leading to rapid monocyte activation and pro-inflammatory cytokine release. The identification of the molecular mechanisms that underpin this novel one-step activation pathway might provide new opportunities for therapeutic intervention in human inflammatory disorders.

## Methods

### Monocyte isolation and stimulation

Peripheral blood mononuclear cells were isolated by Ficoll-Hypaque density gradient centrifugation of buffy coats obtained from anonymous blood donors (Blood Bank of National University Hospital, Singapore; NUS-IRB 12-044E). Monocytes were isolated by negative selection using Monocyte isolation Kit II (Miltenyi Biotech) following the manufacturer's instructions. Monocyte purity was assessed by flow cytometry using a PE-labelled anti-human CD14 antibody (clone 61D3, eBioscience) and was routinely >90%. Monocytes were cultured in plates of 96 wells (1.5 × 10^5^ cells per well) or 24 wells (1 × 10^6^ cells per well) in RPMI 1640-Glutamax medium supplemented with 3% human AB serum, 100 U ml^−1^ penicillin, 100 μg ml^−1^ streptomycin, and 10 mM HEPES (all from Gibco). In some cases, monocytes were pretreated with the specific inhibitors (Z-YVAD-fmk (1–20 μM), LEVD-fmk (1–20 μM), diphenyleneiodonium (DPI, 10 μM) (all from Enzo Lifesciences), R406 (0.5–2 μM, Selleck), dynasore (40–80 μM, Sigma-Aldrich), IP3 receptor antagonist 2-APB (50–100 μM, Calbiochem) or LPS antagonist LPS-RS from *Rhodobacter sphaeroides* (10 μg ml^−1^) for 1 h before the addition of LPS (*Escherichia coli* O55:B5, 10 ng ml^−1^). ATP (1 mM, Sigma-Aldrich) was added for the final 30 min of 5 h incubation with LPS. *E. coli* LPS-Re R515 and lipid A were used at 10 ng ml^−1^.

For IFNAR neutralization, monocytes were pre-treated with a neutralizing antibody against human IFN-α/β receptor chain 2 (anti-hIFNAR, clone MMHAR-2, 5 μg ml^−1^, PBL InterferonSource) or its isotype control (mouse IgG2a, 5 μg ml^−1^, Biolegend) for 1 h before stimulation with LPS alone (10 ng ml^−1^), human recombinant IFN-β alone (250 U ml^−1^, PBL InterferonSource) or a combination of the two for a total of 5 h duration.

### Western blotting

Total cell lysates were prepared using Laemmli buffer containing 5% β-mercaptoethanol. Protein concentrations were determined using Nanodrop ND1000 (Thermo Scientific). Proteins (20–50 μg) were boiled, separated on SDS–PAGE, and transferred onto PVDF membranes (Bio-Rad Laboratories). Membranes were blocked with 5% non-fat dry milk in phosphate-buffered saline (PBS) with 0.1% Tween 20 (PBST, Sigma-Aldrich) for 1 h at room temperature, and incubated overnight at 4 °C with the following primary antibodies diluted 1:1,000 in 5% BSA/PBST; anti-NLRP3 (#AG-20B-0014-C100, Adipogen), anti-IL-1β (#2022), anti-total Syk (#2712), anti-caspase-1 (#4199), anti-caspase-5 (#4429), anti-caspase-4 (#4450) (all from Cell Signaling) and anti-GAPDH (#MAB374, Millipore). For phospho-Syk detection, membranes were blocked and incubated with the primary antibody (#2710S, Cell Signaling) in 5% BSA in Tris-buffered saline with 0.1% Tween 20 (TBST). Densitometry analysis was performed using ImageJ software and the data were normalized against GAPDH. Images have been cropped for presentation. Full-size blot images shown in Figs 1, 2, 3, 4, 5, 6, 7 and Supplementary Figs 1, 5, 8 and 9 are included in Supplementary Fig. 11.

### Cytokine measurements

Human IL-1β and IL-6 (both from Biolegend), as well as IL-1α (R&D Systems), were measured in cell-free supernatants by ELISA.

### Real-time quantitative PCR

RNA was isolated using the RNeasy method (Qiagen) and treated with DNAse I (Promega). Quantitative real-time PCR was performed in triplicate using Go Taq qPCR Master Mix (Promega) with the following validated SYBR Green primer pairs: ISG54 forward 5′-GGAGGGAGAAAACTCCTTGGA-3′, reverse 5′-GGCCAGTAGGTTGCACATTGT-3′; ISG56 forward 5′-TCAGGTCAAGGATAGTCTGGAG-3′, reverse 5′-AGGTTGTGTATTCCCACACTGTA-3′; CASP1 forward 5′-GGAAACAAAAGTCGGCAGAG-3′, reverse 5′-ACGCTGTACCCCAGATTTTG-3′^57^; CASP4 forward 5′-AAGAGAAGCAACGTATGGCAGGAC-3′, reverse 5′-GGACAAAGCTTGAGGGCATCTGTA-3′; CASP5 forward 5′-GGTGAAAAACATGGGGAACTC-3′, reverse 5′-TGAAGAACAGAAAGCAATGAAGT-3′; NLRP3 forward 5′-TGGCTGTAACATTCGGAGATTG-3′, reverse 5′-GAAGTCACCGAGGGCGTTGT-3′; GAPDH forward 5′-CCACATCGCTCAGACACCAT-3′, reverse 5′-GGCAACAATATCCACTTTACCAGAGT-3′. Amplification was performed using a 7500 real-time PCR system (Applied Biosystems) and the relative expression level of each gene was evaluated using the 2^−ΔCt^ or 2^−ΔΔCt^ methods, as indicated. Values are normalized for the expression of the housekeeping gene (GAPDH) and the Ct value of untreated (UT) or control (CTRL) siRNA was used as a calibrator.

### Small interfering RNA-mediated knockdown

Monocytes (5–10 × 10^6^) were nucleofected with 300 nM siRNA according to the manufacturer's instruction (Amaxa Nucleofector Technology, program Y001). Transfected monocytes were incubated for 36 h with 20 ng ml^−1^ IFN-γ and then stimulated with LPS alone (10 ng ml^−1^ for 8 h). The following validated siRNAs were used: NLRP3, 5′-ACCGCGGUGUACGUCUUCUUCCUUU-3′^58^ (AITbiotech); CASP5, 5′-AUAGAACGAGCAACCUUGACAA-3′ and 5′-CUACACUGUGGUUGACGAAAA-3′ (AITbiotech); CASP4, ON-TARGETplus SMART POOL Human Casp4 SiRNA (L-004404-00-0005, Dharmacon): 5′-CUACACUGUGGUUGACGAA-3′, 5′-CCAUAGAACGAGCAACCUU-3′, 5′-CAGCAGAAUCUACAAAUAU-3′, 5′-CGGAUGUGCUGCUUUAUGA-3′.

### ROS measurement

Monocytes were seeded into black 96-well plates (1.5 × 10^6^ cells per well) and rested for ≥2 h. The Syk inhibitor R406 (0.5–2 μM) or vehicle control DMSO was added 30 min before LPS stimulation. The cell-permeable probe H2DCF-DA (10 μM, Enzo Lifesciences) was immediately added and fluorescence was measured every 10 min using a microplate fluorimeter (TECAN; excitation, 480 nm; emission, 530 nm). For each condition, values in triplicate were normalized to the average reading of the untreated control.

### Calcium mobilization assay

Cells were plated into black 96-well plates (1.5 × 10^5^ cells per well) and rested overnight at 37 °C. Cells were incubated for 45 min in the dark with 100 μl of Hanks' balanced salt solution (HBSS) containing HEPES, probenecid (2.5 mM) and Fluo4-NW (20 mM, Invitrogen). In some experiments, cells were pre-incubated with R406 (0.5 μM) or U73122 (3 μM) for 1 h before addition of Fluo4-NW. Fluorescence was monitored before and after the injection of HBSS, LPS (1 μg ml^−1^) or ATP (1 mM) using a Victor4 plate reader (Perkin Elmer; excitation, 485 nm; emission, 535 nm). All experiments were performed at 37 °C. Fluorescence (F) was normalized against the baseline acquired at the time of the stimulation (F0).

### Monocyte-derived DCs and macrophages culture

Monocytes (>95% pure) were positively selected using the CD14^+^ monocyte isolation kit (Miltenyi Biotec) and then transferred into 6-well plates (2 × 10^6^ cells per well) in RPMI 1640-Glutamax medium, supplemented with 10% FBS, 100 U ml^−1^ penicillin, 100 mg ml^−1^ streptomycin, 1 mM sodium pyruvate, and MEM non-essential amino acids (MEM-NEA) (all from Gibco). DCs were generated by culturing monocytes with recombinant human GM-CSF (50 ng ml^−1^, Miltenyi Biotec) and IL-4 (10 ng ml^−1^, R&D Systems), while macrophages were differentiated with recombinant human M-CSF (10 ng ml^−1^, R&D Systems). On day 3, the cytokine-supplemented medium was refreshed. On day 7, DCs and macrophages were plated into fresh plates of 96 wells (1.5 × 10^5^ per well) or 24 wells (1 × 10^6^ cells per well) in complete medium and stimulated for 5 h with 1 μg ml^−1^ *E. coli*-derived LPS (serotype O55:B5) either alone or in combination with 1 mM ATP added to the culture for the last 30 min.

### Cell death measurement

Lactate dehydrogenase (LDH) was measure in cell-free supernatants following the manufacturer's instruction (Promega). For the Annexin V/7-Aminoactinomycin D (7-ADD) staining, monocytes were treated with LPS (10 ng ml^−1^) for the indicated time and subsequently stained using APC-conjugated Annexin V and 7-AAD (both from BD Pharmingen) according to the manufacturer's protocol. Data were acquired on a LSR II flow cytometer (BD Biosciences) for analysis using FlowJo software (TreeStar).

### Immunofluorescence and confocal microscopy

Monocytes were plated in μ-Slides (Ibidi) and stimulated with LPS (10 ng ml^−1^, Enzo Lifesciences) or with biotinylated LPS (10 μg ml^−1^, Invivogen) for the indicated times. Where indicated, R406 (2 μM) or dynasore (40 μM) were added 30 min before stimulation with LPS. Cells were fixed in 2% paraformaldehyde (Polysciences) for 10 min at room temperature and then permeabilized with saponin (0.1% in PBS, 0.2% gelatin and 5 mg ml^−1^ BSA). Cells were stained with CD14-FITC (BD Biosciences), Rab5 and LAMP-1 (both from Cell Signaling), together with DAPI, and imaged on a FV1000 Olympus confocal microscope using a × 100 objective with an additional zoom of 2. Colocalization was quantified using the Pearson's correlation coefficient calculated by Imaris (Bitplane).

### LPS internalization assay

Monocytes were stimulated with biotinylated LPS (10 μg ml^−1^, Invivogen) for the indicated times. Membrane-bound LPS was detected by incubation with AlexaFluor 450-conjugated streptavidin (1:200, eBioscience). After staining, cells were rinsed extensively with PBS to remove excess streptavidin, and then fixed and permeabilized with BD Cytofix/Cytoperm buffer. Intracellular LPS was stained using APC-conjugated streptavidin (1:200, eBioscience) and data were acquired on a LSR II flow cytometer (BD Biosciences) for analysis using FlowJo software (TreeStar).

### Mass spectrometry analysis

To identify caspase-5 cleavage sites prior trypsin digestion, recombinant caspase-5 was labelled for 1 h with Tandem Mass Tags (TMT, Pierce) in order to modify primary free amines (N-terminal) as previously described^59^. Labelling reaction was quenched with 1 M Tris HCl pH 7.4 (15 min, RT) followed by protein denaturation in 50% trifluoroethanol (TFE), 50 mM triethylammoniumbicarbonate (TEAB) pH 8.5. Reduction was done with 20 mM Tris(2-carboxyethyl)phosphine (TCEP) (20 min, 55 °C) and alkylation with 55 mM chloroacetaldehyde (CAA; 20 min, RT) followed by trypsin digestion (18 h, 37 °C) and subsequent acidification with 1% trifluoroacetic acid (TFA). Following desalting on STAGEtips^60^ peptides were separated and analysed by reverse phase liquid chromatography (RP-LC) on Dionex 3000 HPLC system (Thermo Scientific) coupled with Q-Exactive mass spectrometer (Thermo Scientific) in a 100 min gradient of solvent A (0.5% CH3COOH in water) and solvent B (80% MeCN, 0.5% CH3COOH in water). Raw spectra were processed by Proteome Discoverer 1.4 (Thermo Scientific). Obtained peak list files (.mgf) were searched using Mascot 2.5.1 (Matrix-Science) with Uniprot Human database using semi-tryptic or unspecific cleavage with the following parameters: fixed modification, carbamidomethyl cysteine; variable modifications, oxidation on methionine; acetylated N-terminal protein, TMT label or dimethyl labelling. Since TMT blocks also lysine residues cleavage we used 4 missed cleavages option, MS accuracy 20 p.p.m., MS/MS accuracy 0.03 Da.

### Statistical analysis

Data were analysed using Prism 6 software (GraphPad) and statistical significance was determined using the one-sample *t*-test.

## Additional information

**How to cite this article:** Viganò, E. *et al*. Human caspase-4 and caspase-5 regulate the one-step non-canonical inflammasome activation in monocytes. *Nat. Commun.* 6:8761 doi: 10.1038/ncomms9761 (2015).

## Supplementary Material

Supplementary Information Supplementary Figures 1-11
