# Supplementary material for: Human caspase-4 and caspase-5 regulate the one-step non-canonical inflammasome activation in monocytes
Source: Nat Commun. 2015 Oct 28;6:8761. doi: 10.1038/ncomms9761 (PMC4640152; doi:10.1038/ncomms9761)
Supplement: Supplementary Information — Supplementary Figures 1-11 [file ncomms9761-s1.pdf]

SUPPLEMENTARY FIGURES

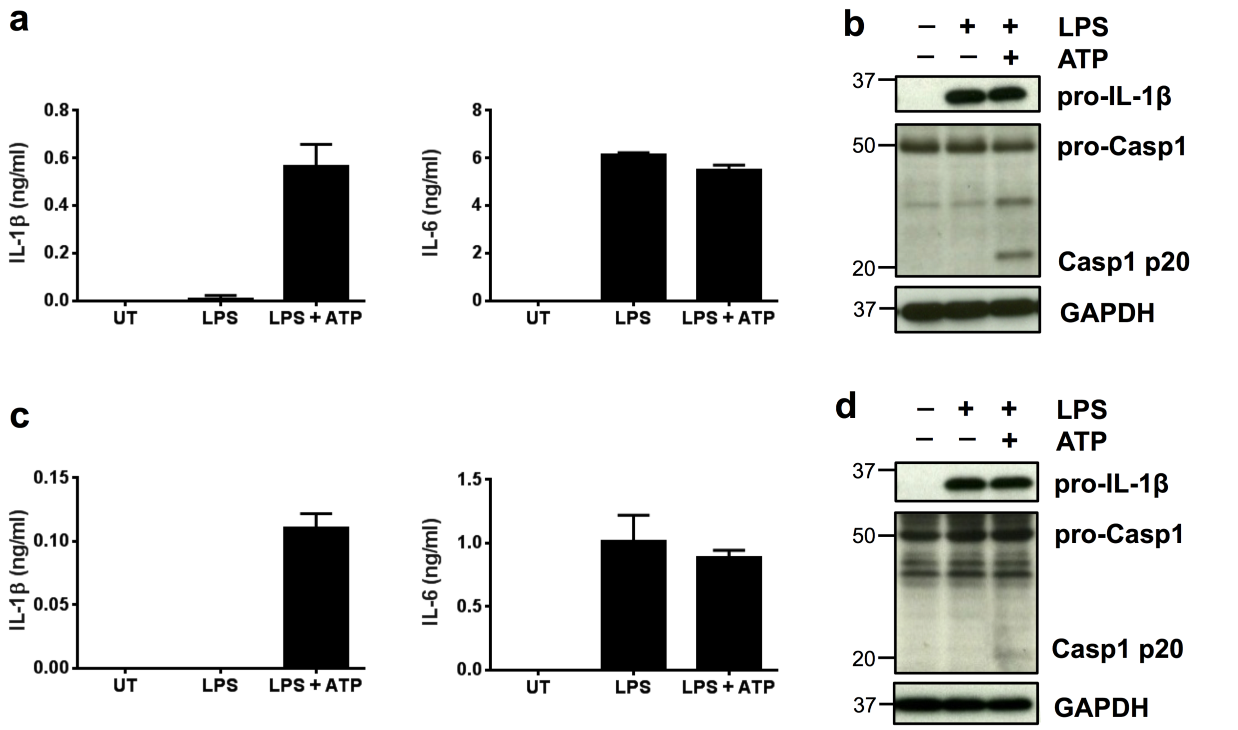

Supplementary Figure 1. Dendritic cells and macrophages require a second stimulus to activate the NLRP3 inflammasome. ELISA (a,c) and western blot (b,d) analyses of dendritic cells (a,b) and macrophages (c,d) cultured in medium alone, or stimulated with LPS in the presence or absence of ATP. Secretion of IL-1β and IL-6 cytokines (a,c) was determined in supernatants, and pro-IL-1β, pro-caspase-1, and caspase-1 p20 expression levels (b,d) in cell lysates. GAPDH was used as the loading control. Graphs show the mean ± standard deviation of triplicate wells and are representative of three independent experiments.

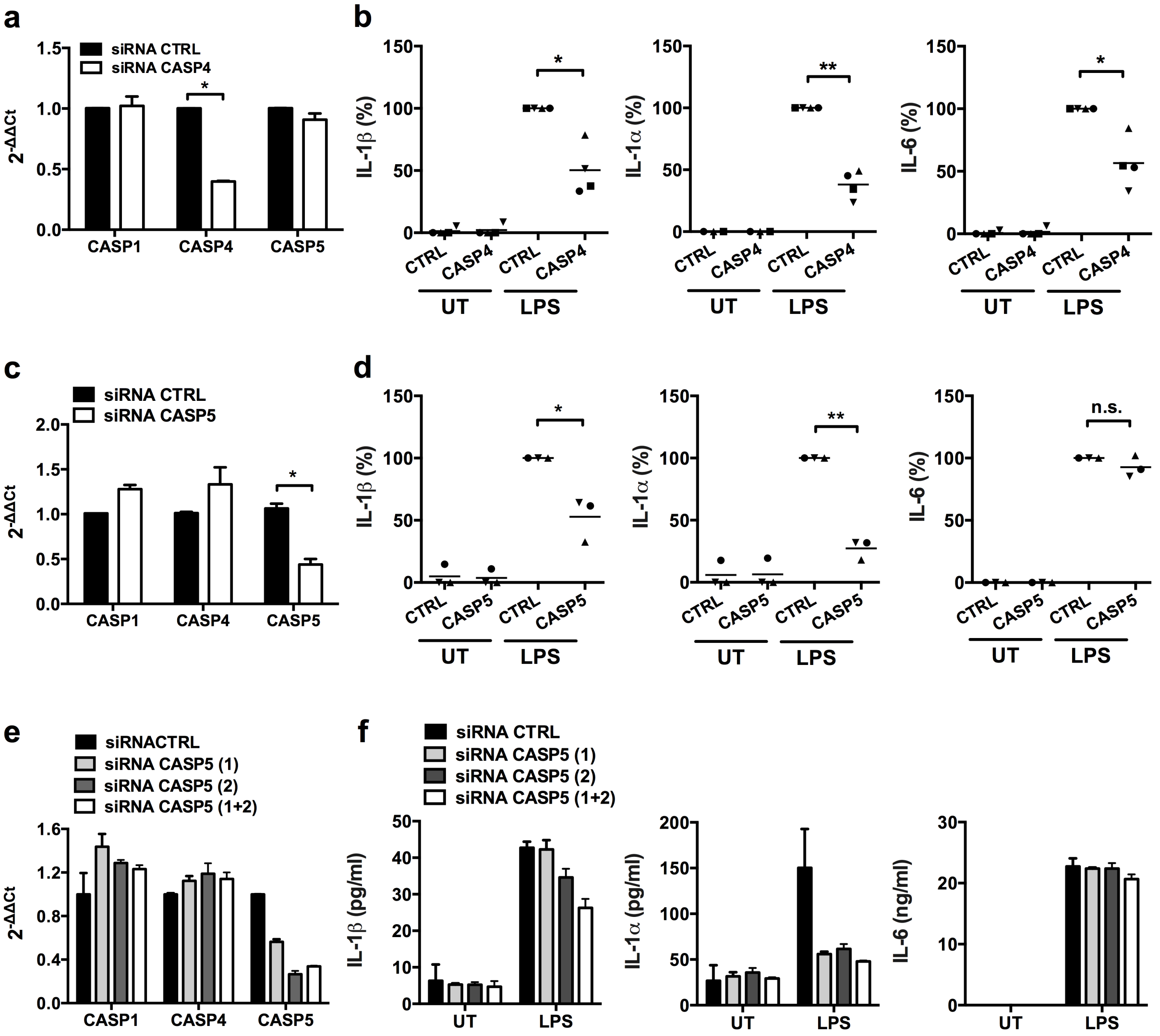

Supplementary Figure 2. Caspase-4 and caspase-5 regulate the release of IL-1β and IL-1α from monocytes after LPS treatment. (a,c,e) Quantitative PCR analysis of caspase-1, -4 and -5 expression determined in monocytes after nucleofection of caspase-4 (a) or caspase-5 (c,e) siRNA duplexes. (b,d,f) IL-1β, IL-1α and IL-6 release from unstimulated and LPS-stimulated monocytes in which caspase-4 (b) or caspase-5 expression (d,f) was knocked down. Graphs show the mean ± standard deviations normalized as percent of the LPS-stimulated siRNA control. Each value represents monocyte response of independent donors and significance evaluated using one sample t-test. * p-value < 0.05, ** p-value < 0.01.

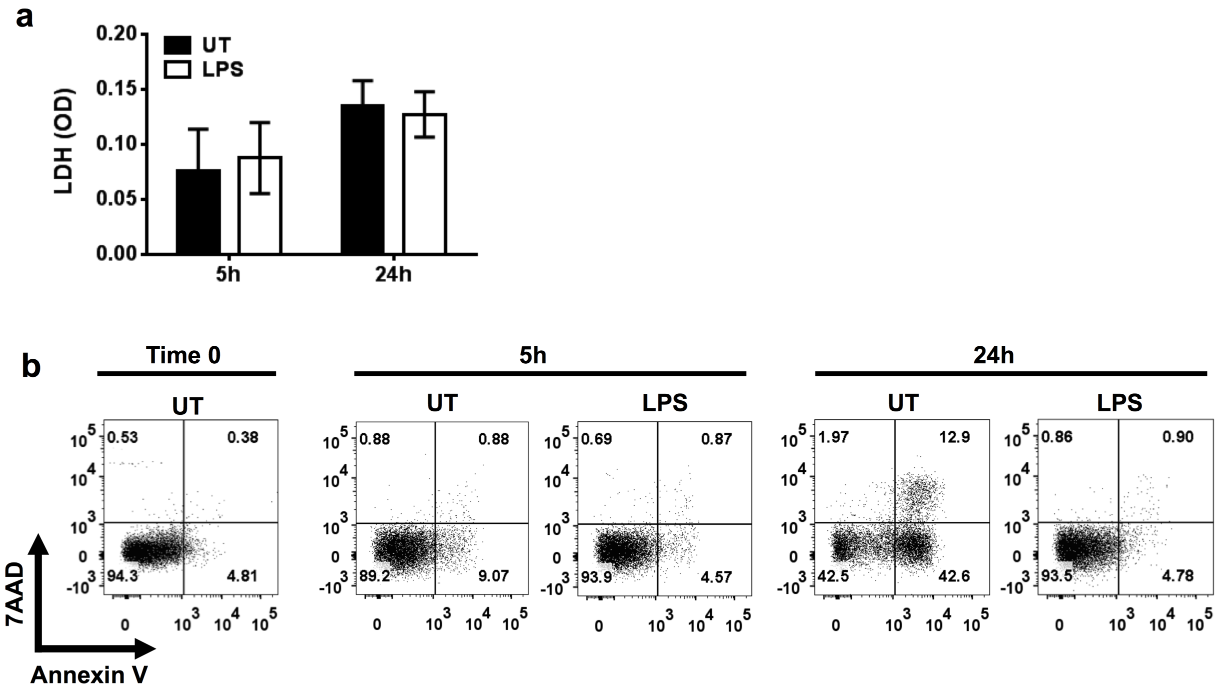

Supplementary Figure 3. Human monocytes do not undergo cell death following LPS stimulation. Cytotoxicity was determined by LDH released in cell-free supernatants (a) or as annexin V/7-AAD staining (b) of monocytes left untreated or stimulated with LPS for 5 and 24h. Graph a shows the mean ± standard deviation of three independent experiments.

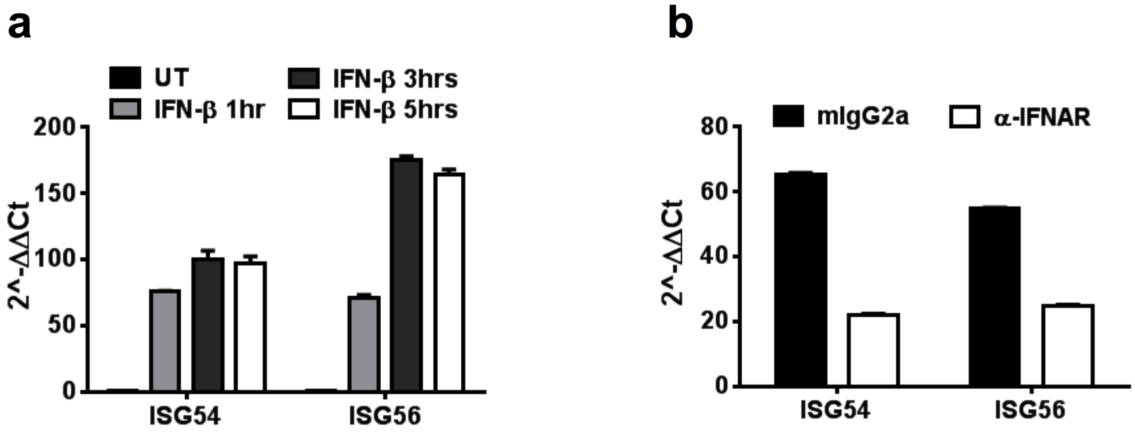

Supplementary Figure 4. Effect of IFNAR blocking on expression of IFN stimulated genes. ISG54 and ISG56 expression were assessed by quantitative real-time PCR in monocytes treated with IFN-β for the indicated times (a) or in monocytes that were pre-treated with a neutralizing anti-IFNAR antibody (α-IFNAR) or isotype-matched control (mIgG2a) prior to IFN-β stimulation (b). Graphs show the mean ± standard deviation of triplicate wells and are representative of three independent experiments.

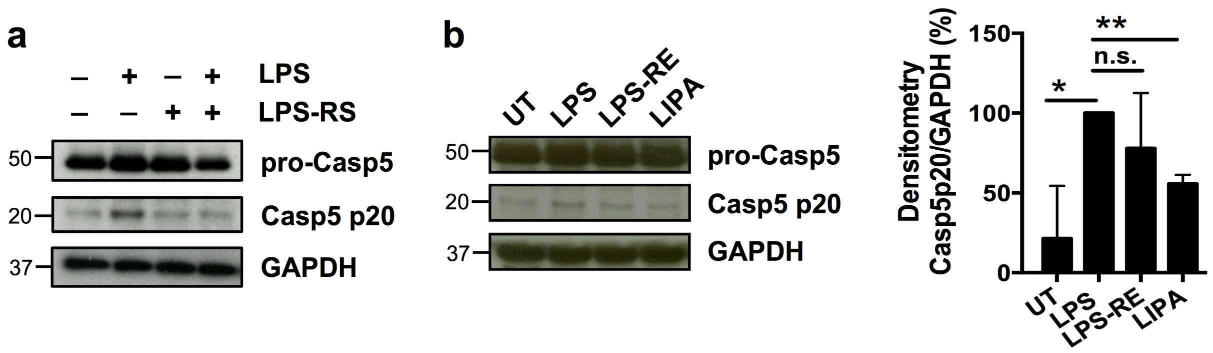

Supplementary Figure 5. Caspase-5 processing requires TLR4 signaling. (a) Monocytes were treated with the LPS antagonist LPS-RS from *Rhodobacter sphaeroides* (10 µg/ml) for 1h prior to LPS stimulation. Caspase-5 processing was measured in lysates by western blot. (b) Caspase-5 processing in monocytes stimulated with LPS, LPS-RE or lipid A (LIPA) was assessed by western blot (left). Densitometry analysis was performed and caspase-5 p20 expression is shown on the right as mean ± standard deviation of three independent experiments and significance evaluated using a one sample t-test. * p-value < 0.05, ** p-value < 0.01, n.s. not significant.

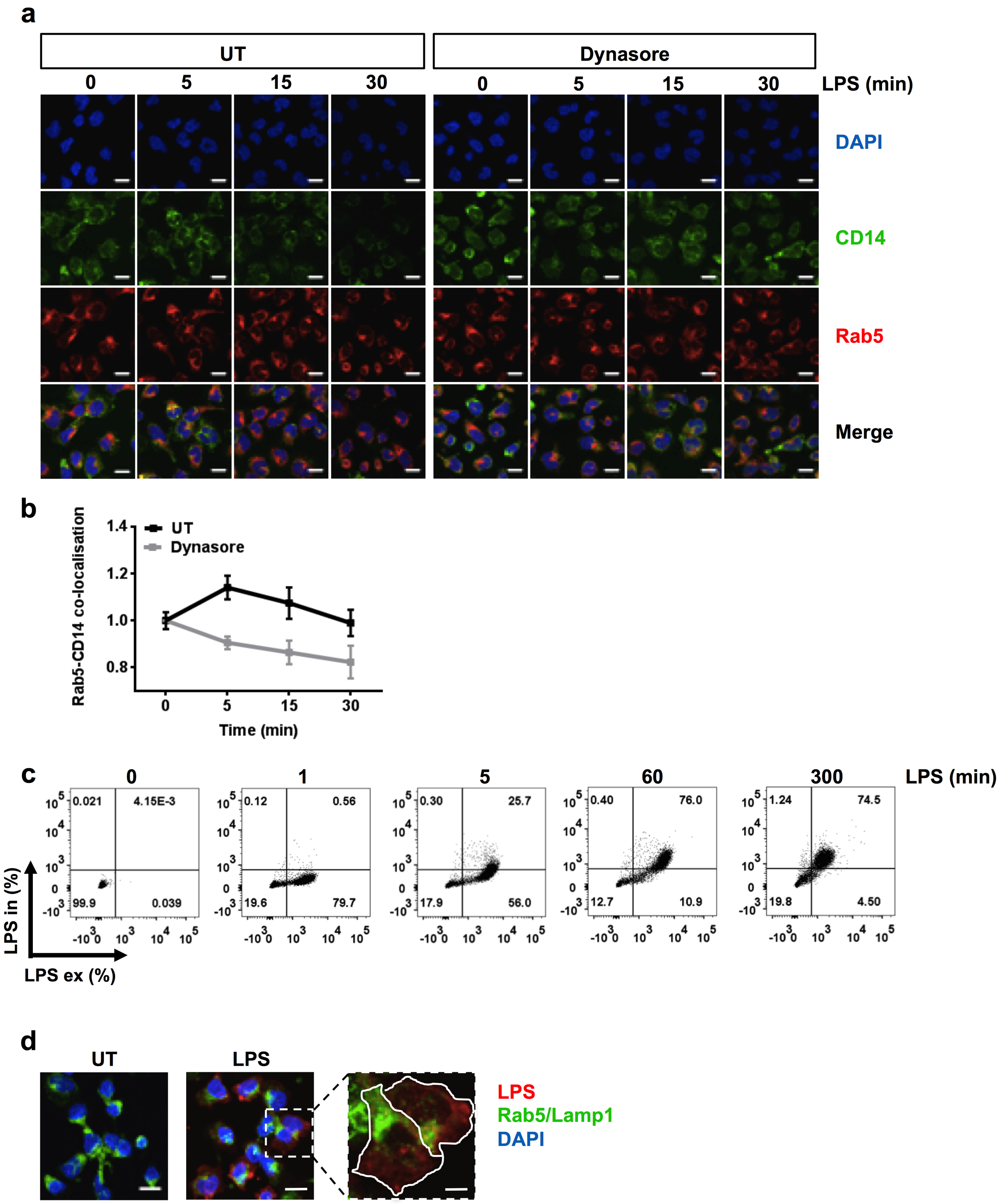

Supplementary Figure 6. Endosomal re-localization of CD14 in LPS-stimulated monocytes. (a,b) CD14/Rab5 co-localization in monocytes that were treated or not with dynasore prior to LPS stimulation. Co-localization was evaluated using images from three independent experiments for each time point, and Pearson's correlation coefficient was calculated by averaging values from ~100 cells across five imaging fields. Scale bar, 10 µm. (c) Membrane-bound LPS (LPS ex) and intracellular LPS (LPS in) were determined by flow-cytometry analysis of monocytes that were treated or not with biotinylated LPS for the indicated times. Inset numbers indicate the proportion of positive cells in each quadrant. (d) Z-stack projections of the focal planes of monocytes that were untreated (UT) or stimulated with LPS for 1h (LPS in red, Rab5/Lamp1 early and late endocytic markers in green, and nuclear staining in blue). Scale bar, 10 µm.

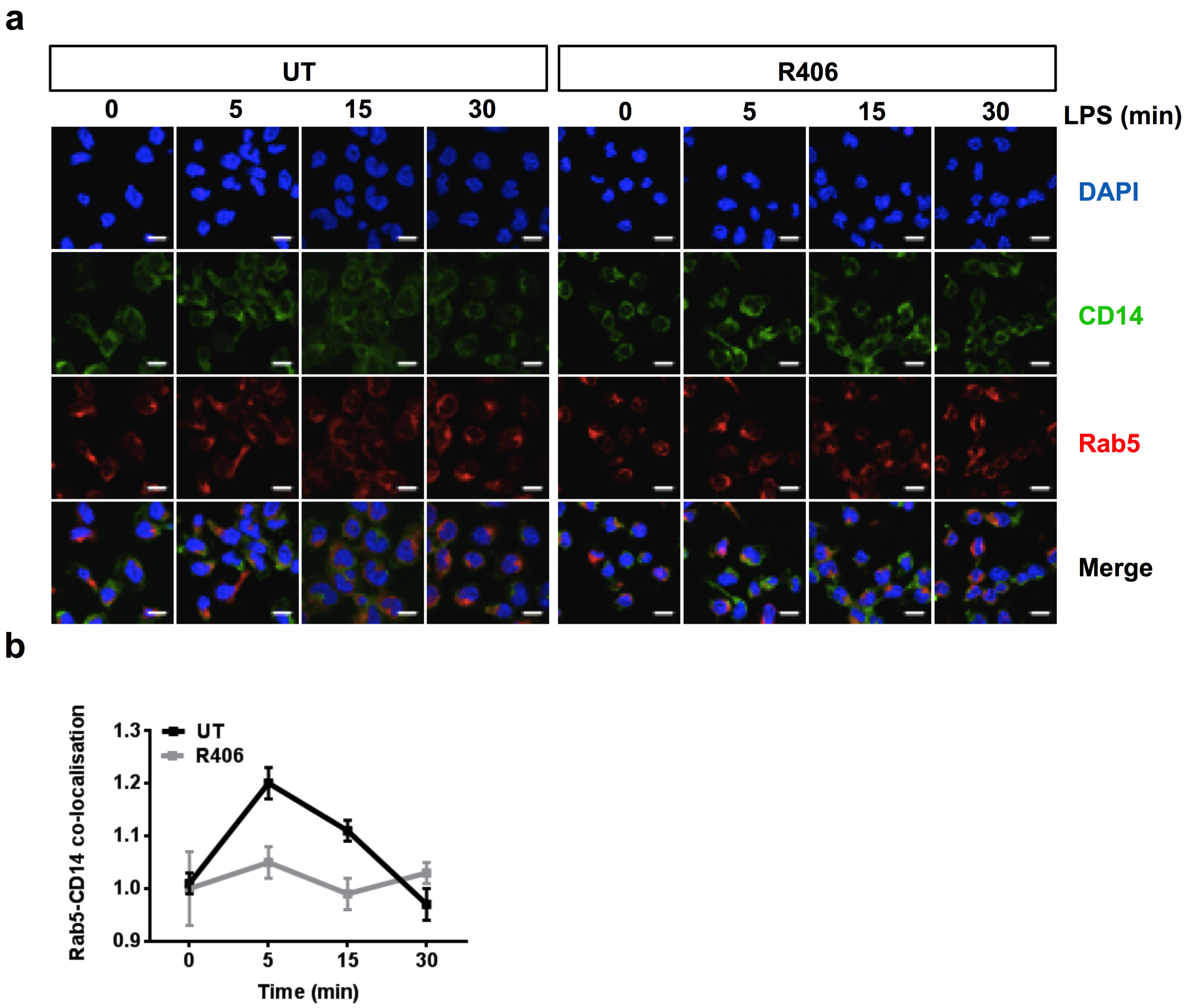

Supplementary Figure 7. Endosomal CD14 re-localization depends on Syk. (a) Co-localization of CD14 (green) and Rab5 (red) in monocytes that were pre-treated with the Syk inhibitor R406 for 30min and then stimulated with LPS for the indicated times. Nuclei were stained using DAPI (blue). Scale bar, 10 µm. (b) Co-localization was evaluated using images from three independent experiments for each time point, and Pearson's correlation coefficient was calculated by averaging values from ~100 cells across five imaging fields.

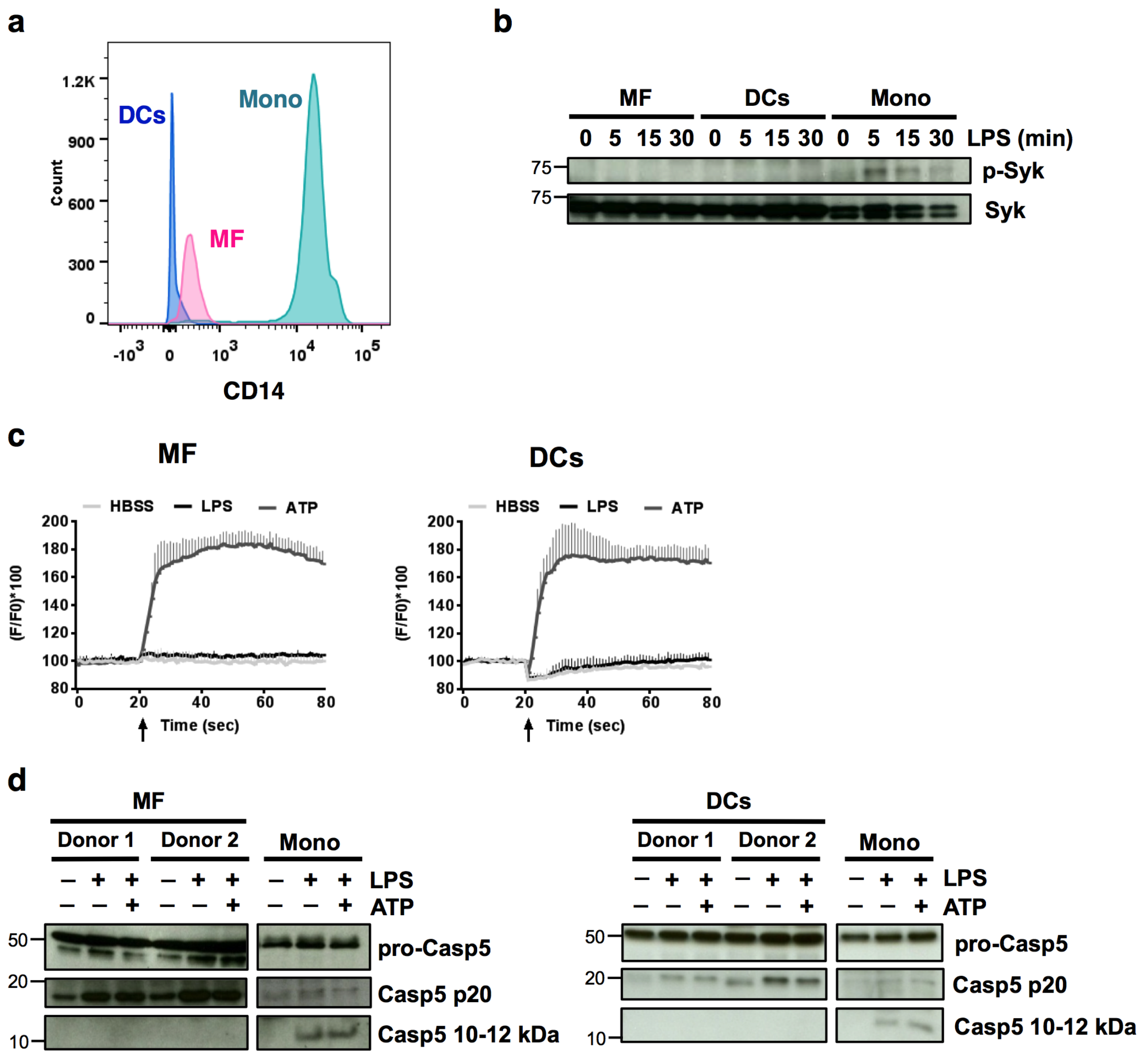

Supplementary Figure 8. Defective Syk activation and Ca^{2+} mobilization in macrophages and DCs following LPS stimulation. (a) CD14 expression in monocytes (mono), macrophages (MF) and dendritic cells (DCs) differentiated in culture from the same donor. (b) Syk phosphorylation was assessed in MF, DCs and monocytes upon LPS stimulation for 5, 15 and 30min by western blot analysis. (c) Mobilization of Ca^{2+} in MF (left) and DCs (right) after stimulation with LPS (1 µg/ml) or ATP (1 mM). Arrows indicate the timing of stimuli addition. (d) Caspase-5 processing in MF (left), DCs (right) and monocytes stimulated with LPS, LPS/ATP or left untreated. GAPDH was used as the loading control. Graphs show the mean ± standard deviation of triplicate wells and are representative of three independent experiments.

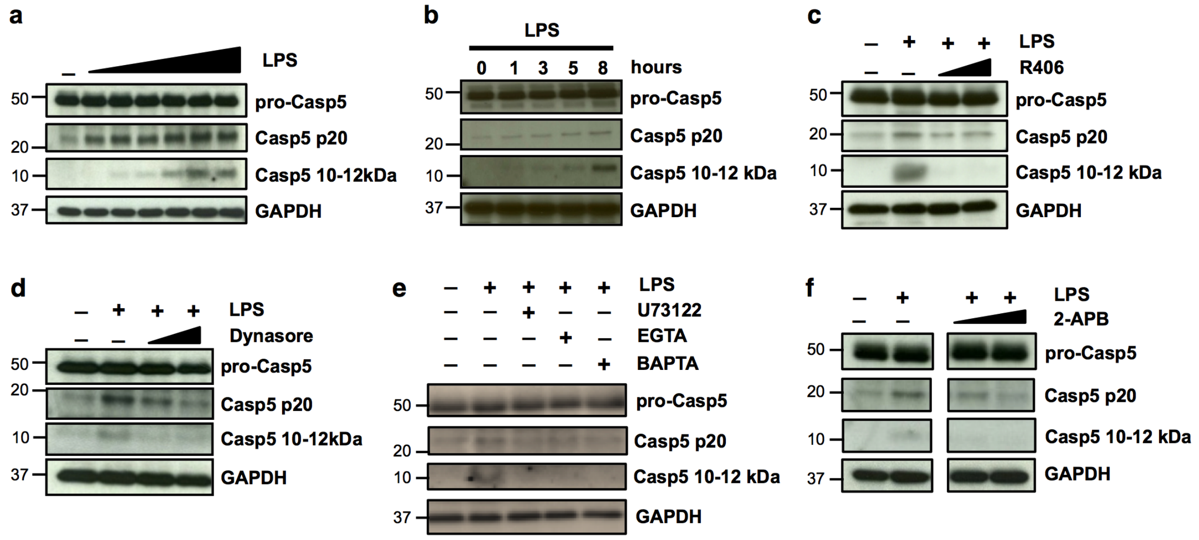

Supplementary Figure 9. Identification of a caspase-5 10-12 kDa fragment associated with LPS response in monocytes. Western blot analyses of caspase-5 processed forms (precursor, p20 subunit and 10-12 kDa) in lysates from monocytes stimulated with different LPS doses (a) and LPS exposure times (b), or in lysates of monocytes pre-treated with Syk inhibitor (c), dynamin inhibitor (d), the PLCγ inhibitor U73122 (e), the Ca^{2+} chelators EGTA and BAPTA (e), or IP3 receptor antagonist (f). GAPDH was used as the loading control. The blots shown here are the same as those shown in Fig. 2d, 2e, 5c, 5d, 7b and 7c.

a

1	MAEDSGKKKR RKNFEAMFKG ILQSGLDNFV INHMLKNNVA GQTSIQTLVP NTDQKSTSVK
61	KDNHKKKTVK MLEYLGKDVL HGVFNYLAKH DVLTLKEEEK KKYYDTKIED KALILVDSLR
	p20
121	KNRVAHQMFT QTLLNMDQKI TSVKPLLQIE AGPPESAEST NILKLCPREE FLRLCKKNHD
181	EIYPIKKRED RRRLALIICN TKFDHLPARN GAHYDIVGMK RLLQGLGYTV VDEKNLTAR***D****
241	***MESVLR***AFAA RPEHKSSDST FLVLMSHGIL EGICGTAHKK KKPDVLLYDT IFQIFNNRNC
	p10
301	LSLKDKPKVI IVQACRGEKH GELWVRDSPA SLALISSQSS ENLEADSVCK IHEEKDFIAF
361	CSSTPHNVSW RDRTRGSIFI TELITCFQKY SCCCHLMEIF RKVQKSFEVP QAKAQMPTIE
421	RATLTRDFYL FPGN

b

Sequence: DMESVLR
D1-TMT6plex (229.16293 Da)
Charge: +2, Monoisotopic m/z: 539.79230 Da (+0.43mmu/+0.79 ppm), MH=: 1078.57732 Da

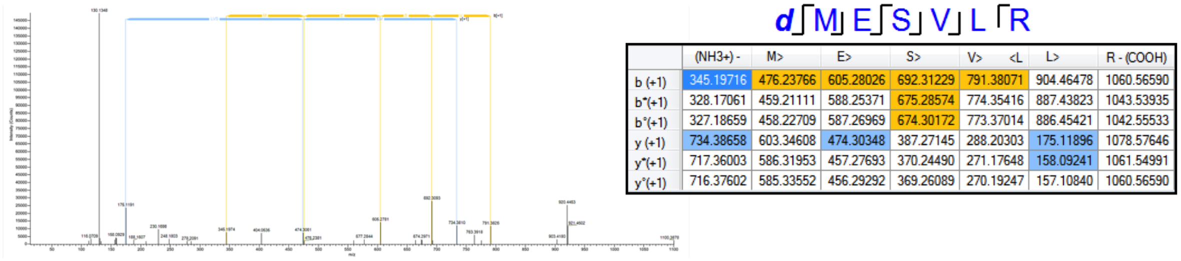

d⌋⌈M⌋ E⌋⌈S⌋ V⌋ L ⌈R

	(NH3+) -	M>	E>	S>	V> <L	L>	R - (COOH)
b (+1)	345.19716	476.23766	605.28026	692.31229	791.38071	904.46478	1060.56590
b*(+1)	328.17061	459.21111	588.25371	675.28574	774.35416	887.43823	1043.53935
b°(+1)	327.18659	458.22709	587.26969	674.30172	773.37014	886.45421	1042.55533
y (+1)	734.38658	603.34608	474.30348	387.27145	288.20303	175.11896	1078.57646
y*(+1)	717.36003	586.31953	457.27693	370.24490	271.17648	158.09241	1061.54991
y°(+1)	716.37602	585.33552	456.29292	369.26089	270.19247	157.10840	1060.56590

Supplementary Figure 10. Alternative proteolytic cleavage of caspase-5. (a) Amino acid sequence of human caspase-5 isoform a. Caspase-5 enzyme is synthesized as a 48 kDa precursor polypeptide, which contains a pro-domain (in black) followed by the 20 kDa subunit (p20, in ochre) joined by a short linker peptide (in black) to the 10 kDa subunit (p10, in blue). An alternative Asp (D)-X cleavage site within the p20 subunit has been identified (D*240). DMESVLR peptide (in green) corresponds to the N-terminus labeled peptide detected by mass spectrometry. L146 (in red) and the surrounded area correspond to the epitope recognized by the anti-caspase-5 antibody. The caspase-5 active site (C315) is highlighted in purple. (b) MS/MS spectrum of peptide DMESVLR (2+) from caspase-5 was extracted and ion series (b, y) were annotated using PD 1.4 with clear N-terminal modification (TMT-reporter ion 130.1348 m/z). Detailed ion masses are shown in the table next to the spectrum.

Fig 1b

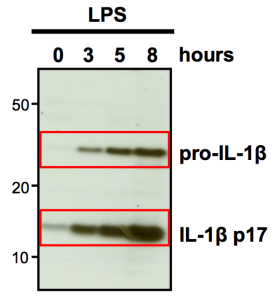

Fig 1c

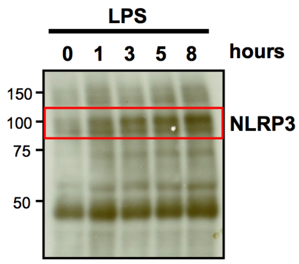

Fig 1d

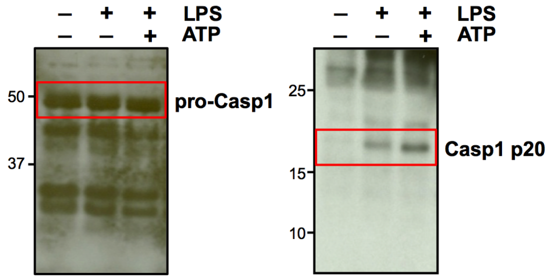

Fig 1g

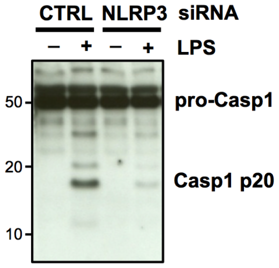

Fig 2c

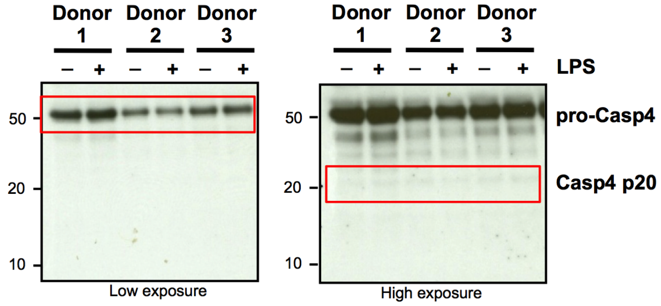

Fig 2d & Suppl. Fig 9a

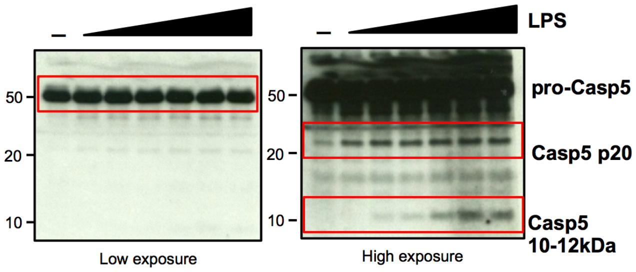

Fig 2e & Suppl. Fig 9b

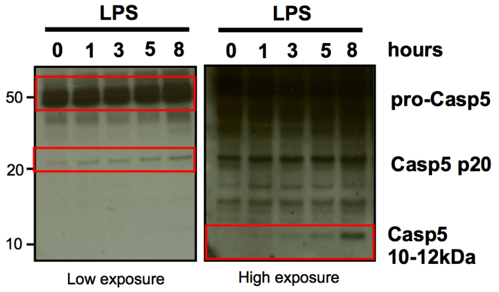

Fig 2f

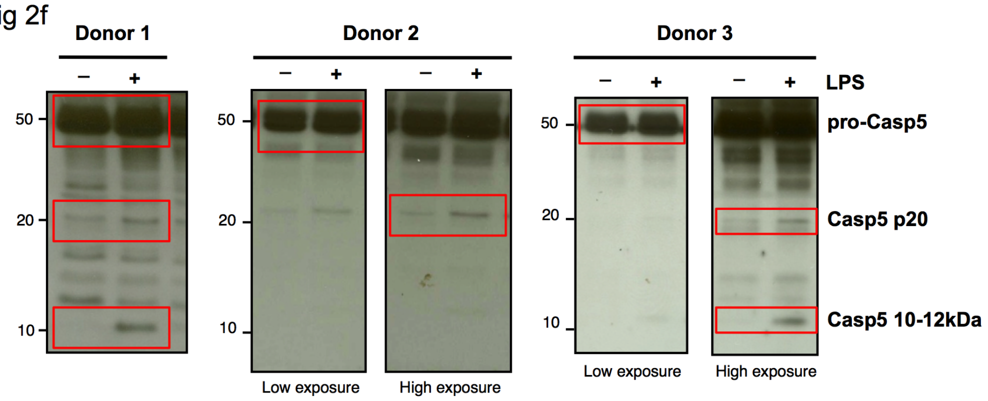

Cont'd

Fig 3b

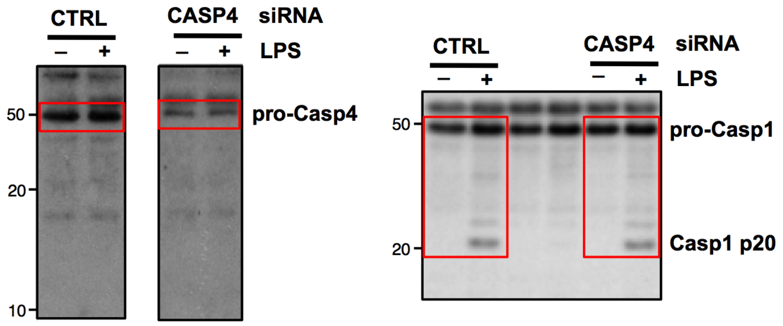

Fig 3d

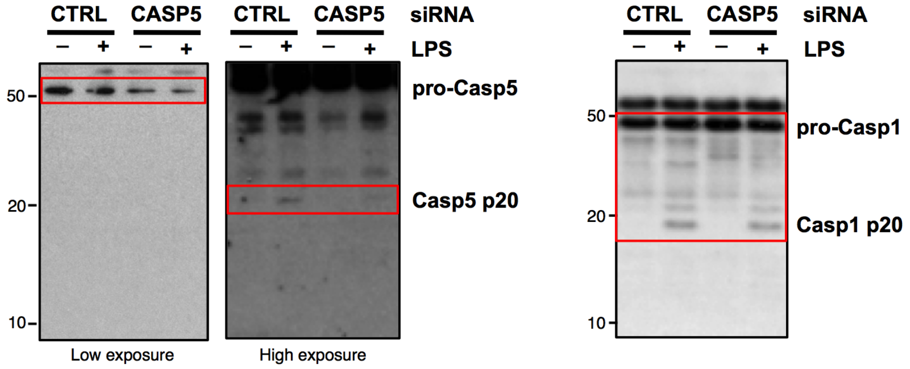

Fig 4b

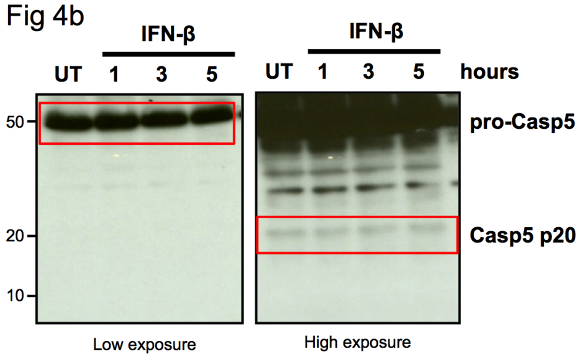

Fig 4d

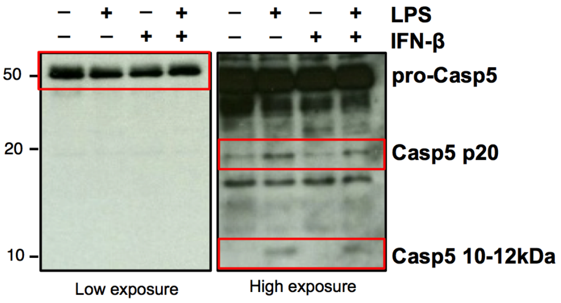

Fig 4f

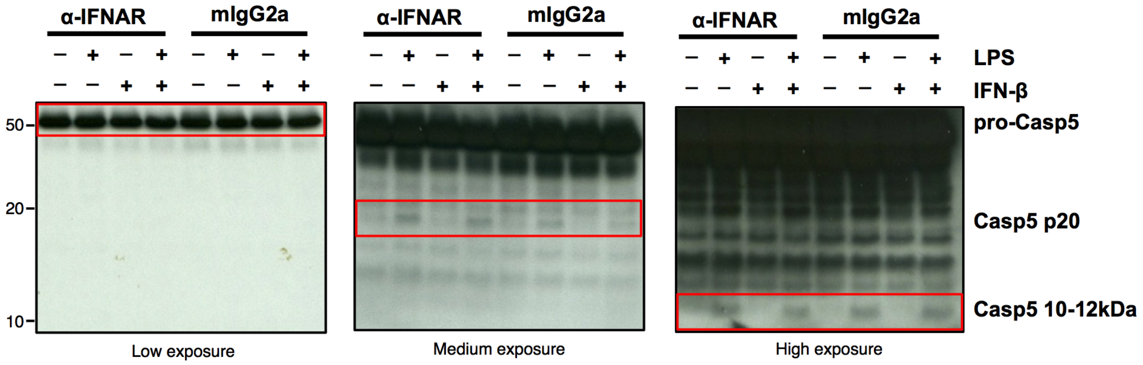

Cont'd

Fig 5a

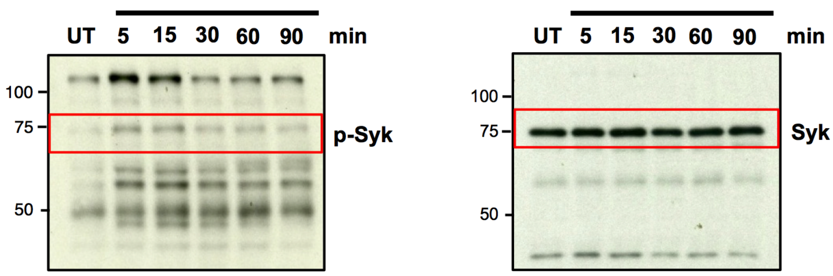

Fig 5b

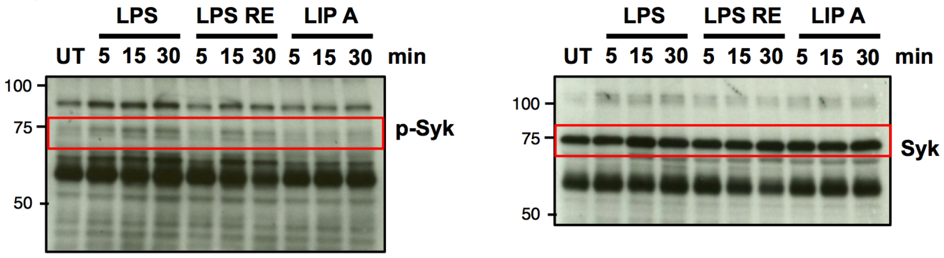

Fig 5c & Suppl. Fig 9c

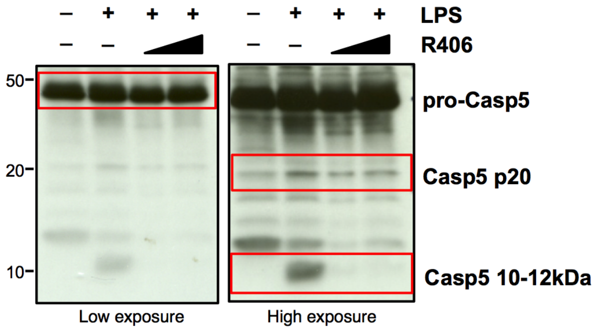

Fig 5d & Suppl. Fig 9d

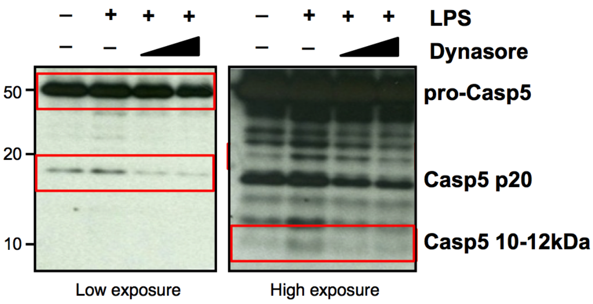

Fig 6b

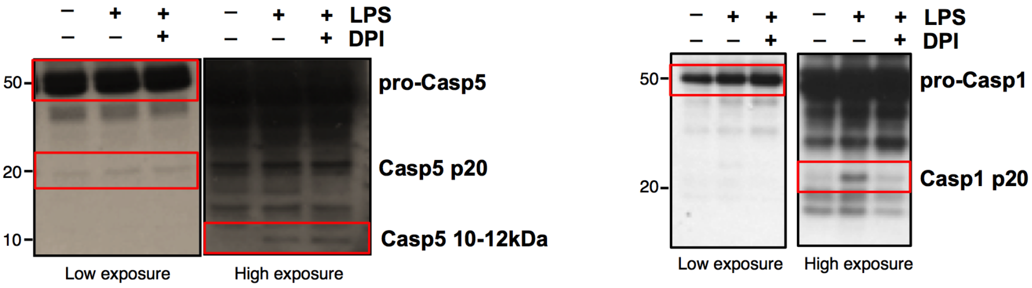

Cont’d

Fig 7b & Suppl. Fig 9e

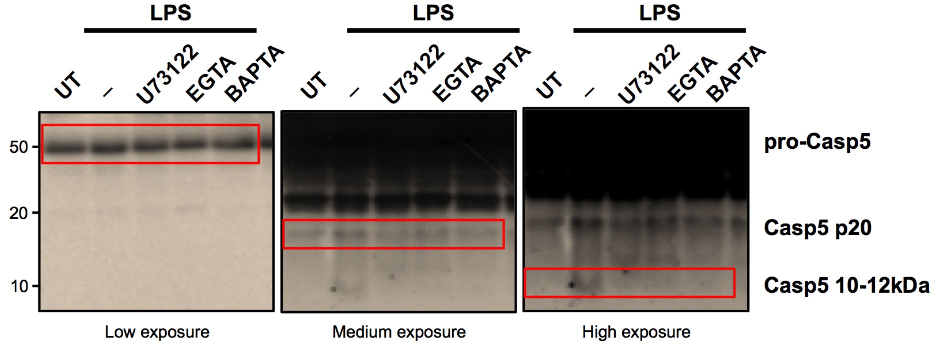

Fig 7c & Suppl. Fig 9f

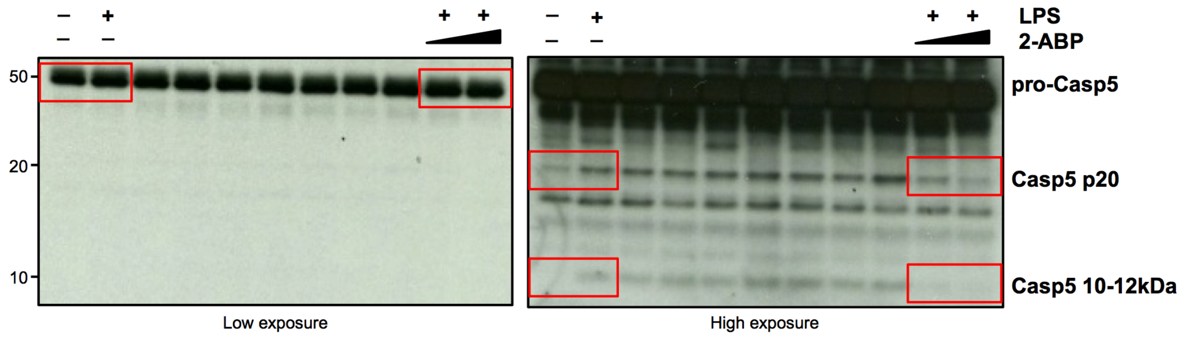

Suppl. Fig 1b

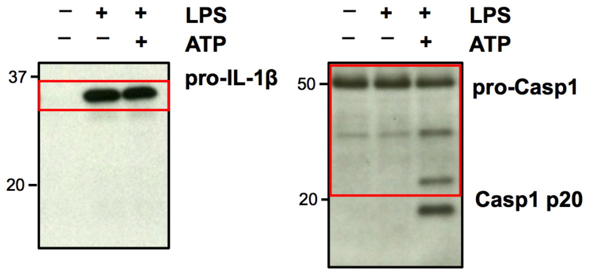

Suppl. Fig 1d

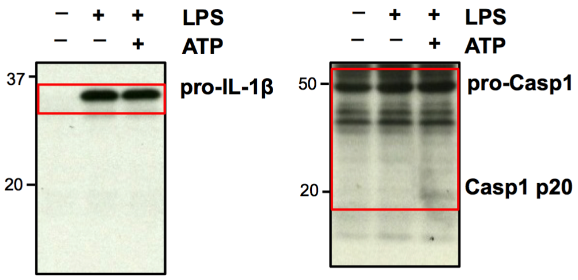

Suppl. Fig 5a

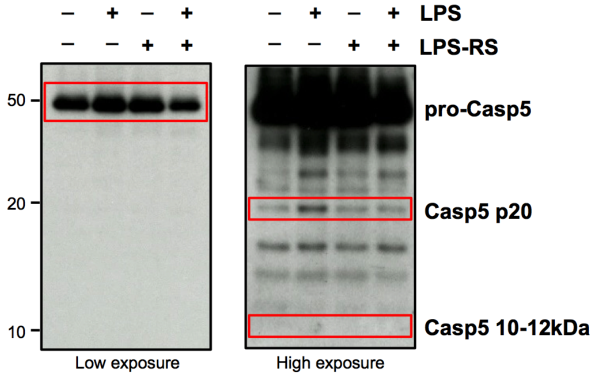

Suppl. Fig 5b

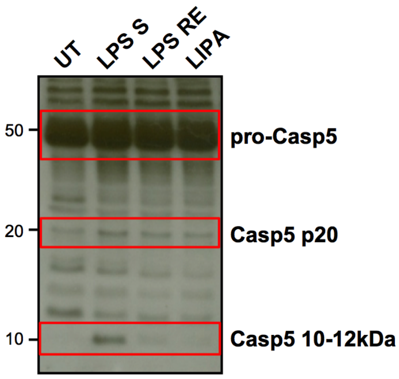

Cont'd

Suppl. Fig 8b

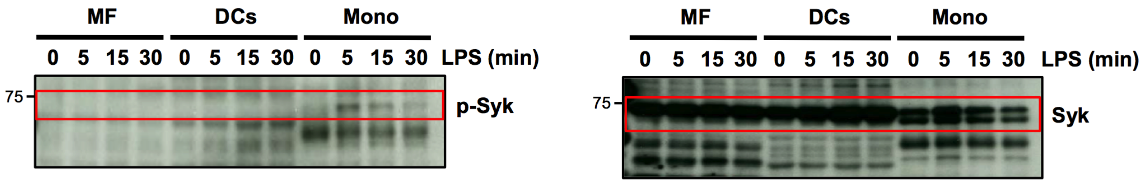

Suppl. Fig 8d

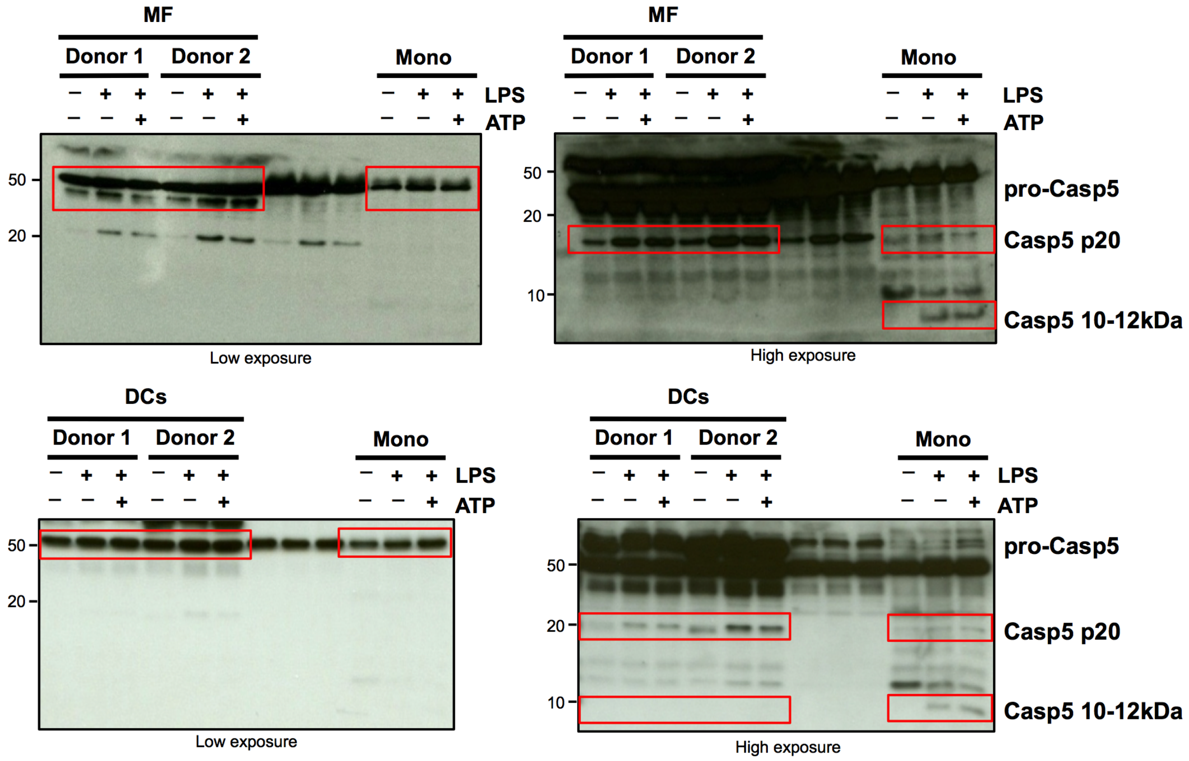

Supplementary Figure 11. Uncropped blots related to the western blots shown in Figures 1-7 and Supplementary Figures 1, 5, 8 and 9. The red boxes highlight the regions of the western blots shown in the figures.
